# Gel‐like inclusions of C‐terminal fragments of TDP‐43 sequester stalled proteasomes in neurons

**DOI:** 10.15252/embr.202153890

**Published:** 2022-04-19

**Authors:** Henrick Riemenschneider, Qiang Guo, Jakob Bader, Frédéric Frottin, Daniel Farny, Gernot Kleinberger, Christian Haass, Matthias Mann, F. Ulrich Hartl, Wolfgang Baumeister, Mark S Hipp, Felix Meissner, Rubén Fernández‐Busnadiego, Dieter Edbauer

**Affiliations:** ^1^ German Center for Neurodegenerative Diseases (DZNE), Munich Munich Germany; ^2^ Department of Molecular Structural Biology Max Planck Institute of Biochemistry Martinsried Germany; ^3^ State Key Laboratory of Protein and Plant Gene Research School of Life Sciences and Peking‐Tsinghua Center for Life Sciences Peking University Beijing China; ^4^ Department of Proteomics and Signal Transduction Max Planck Institute for Biochemistry Martinsried Germany; ^5^ Department of Cellular Biochemistry Max Planck Institute for Biochemistry Martinsried Germany; ^6^ Institute for Integrative Biology of the Cell (I2BC) Université Paris‐Saclay CEA CNRS Gif‐sur‐Yvette France; ^7^ Chair of Metabolic Biochemistry Faculty of Medicine Biomedical Center (BMC) Ludwig‐Maximilians‐Universität Munich Munich Germany; ^8^ Munich Cluster of Systems Neurology (SyNergy) Munich Germany; ^9^ Department of Biomedical Sciences of Cells and Systems University Medical Center Groningen University of Groningen Groningen The Netherlands; ^10^ School of Medicine and Health Sciences Carl von Ossietzky University Oldenburg Oldenburg Germany; ^11^ Department of Systems Immunology and Proteomics Medical Faculty Institute of Innate Immunity University of Bonn Germany; ^12^ Institute of Neuropathology University Medical Center Göttingen Göttingen Germany; ^13^ Cluster of Excellence “Multiscale Bioimaging: from Molecular Machines to Networks of Excitable Cells” (MBExC) University of Göttingen Göttingen Germany; ^14^ Graduate School of Systemic Neurosciences (GSN) Ludwig‐Maximilians‐University Munich Munich Germany

**Keywords:** ALS, neurodegeneration, phase separation, proteasome, TDP‐43, Molecular Biology of Disease, Neuroscience

## Abstract

Aggregation of the multifunctional RNA‐binding protein TDP‐43 defines large subgroups of amyotrophic lateral sclerosis and frontotemporal dementia and correlates with neurodegeneration in both diseases. In disease, characteristic C‐terminal fragments of ~25 kDa ("TDP‐25") accumulate in cytoplasmic inclusions. Here, we analyze gain‐of‐function mechanisms of TDP‐25 combining cryo‐electron tomography, proteomics, and functional assays. In neurons, cytoplasmic TDP‐25 inclusions are amorphous, and photobleaching experiments reveal gel‐like biophysical properties that are less dynamic than nuclear TDP‐43. Compared with full‐length TDP‐43, the TDP‐25 interactome is depleted of low‐complexity domain proteins. TDP‐25 inclusions are enriched in 26S proteasomes adopting exclusively substrate‐processing conformations, suggesting that inclusions sequester proteasomes, which are largely stalled and no longer undergo the cyclic conformational changes required for proteolytic activity. Reporter assays confirm that TDP‐25 impairs proteostasis, and this inhibitory function is enhanced by ALS‐causing TDP‐43 mutations. These findings support a patho‐physiological relevance of proteasome dysfunction in ALS/FTD.

## Graphical abstract

TDP‐25, a C‐terminal fragment of TDP‐43 found in ALS and FTD patients, forms cytoplasmic inclusions with gel‐like properties in primary neurons. Proteasome enrichment and impaired proteostasis support the relevance of proteasome dysfunction in ALS/FTD.

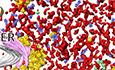

## Introduction

TDP‐43 aggregation is the disease‐defining pathological hallmark in > 90% of patients with amyotrophic lateral sclerosis (ALS) and ~45% of patients with frontotemporal dementia (FTD) (Gao *et al*, 2017; Prasad *et al*, 2019; Tziortzouda *et al*, 2021). However, we still have a limited understanding of the native structure of TDP‐43 inclusions and their role in disease (Gao *et al*, 2017; Chien *et al*, 2021). Predominantly, cytoplasmic neuronal inclusions of TDP‐43 correlate strongly with regional neuron loss in spinal cord, motor cortex, or frontal‐temporal cortical regions (Mackenzie *et al*, 2013). These inclusions are enriched in 25‐35 kDa C‐terminal fragments of TDP‐43 that contain a glycine‐rich low‐complexity region (Neumann *et al*, 2006; Igaz *et al*, 2008). Rare autosomal dominant ALS‐causing mutations in TDP‐43 cluster in this region, although their pathomechanism is still poorly understood, and they have only modest effects *in vitro* and in knockin models (Prasad *et al*, 2019). However, overexpression of the inclusion‐forming 25 kDa C‐terminal fragment ("TDP‐25") triggers neurodegeneration in mice even without disease‐associated mutations (Walker *et al*, 2015).

Distinct biophysical mechanisms can drive inclusion formation in the context of neurodegenerative diseases: (i) formation of highly insoluble amyloid fibrils with cross β‐sheet conformation (Eisenberg & Jucker, 2012), (ii) liquid‐liquid phase separation (LLPS) into highly dynamic liquid‐like droplets (Gomes & Shorter, 2019), which may solidify and adopt amyloid‐like conformations under pathological conditions (Kato *et al*, 2012; Patel *et al*, 2015; Qamar *et al*, 2018). By adopting aberrant conformations, aggregated proteins may engage in toxic cellular interactions (Olzscha *et al*, 2011; Hipp *et al*, 2019). Rapid advances in cryo‐electron microscopy (cryo‐EM) elucidated the structure of amyloid fibrils formed by various disease‐associated aggregating proteins purified from patient tissue at near‐atomic resolution, most recently including TDP‐43 (Creekmore *et al*, 2021; Arseni *et al*, 2022). Cryo‐EM revealed a unique amyloid conformation of TDP‐43 fibrils purified from patients, with little resemblance to fibrillar structures derived from recombinant TDP‐43 C‐terminal region (at pH 4) and shorter synthetic peptide fragments (Guenther *et al*, 2018; Cao *et al*, 2019; Li *et al*, 2021; Arseni *et al*, 2022). Full‐length TDP‐43 has also been reported to form liquid droplets through LLPS *in vitro* (Conicella *et al*, 2016), and RNA‐free TDP‐43 can assemble into so‐called anisosomes consisting of a liquid TDP‐43 shell and a HSP70 core in the nucleus, suggesting that TDP‐43 condensates solidify into fibrils in disease (Yu *et al*, 2021; Arseni *et al*, 2022). ALS/FTD‐causing mutations in *C9orf72*, *OPTN*, *SQSTM1*, *TBK1*, *UBQLN2*, and *VCP* are linked to the ubiquitin‐proteasome system (UPS) and autophagy, which can clear aggregated and phase‐separated proteins, suggesting the proteostasis system is of particular importance at least in genetic ALS/FTD (Gitcho *et al*, 2009; Deng *et al*, 2011; Hipp *et al*, 2019). This view is reinforced by the observation that proteasome inhibition leads to the formation of TDP‐43 anisomes (Yu *et al*, 2021).

Cryo‐electron tomography (cryo‐ET) is a powerful method to visualize neurotoxic inclusions within their native cellular environment complementing the analysis of purified fibrils. Cryo‐ET studies have revealed a striking heterogeneity of ultrastructure and pathomechanisms for neuronal inclusion proteins (Bauerlein *et al*, 2020). In particular, we have used cryo‐ET to show that amyloid‐like poly‐GA inclusions linked to *C9orf72* ALS/FTD disrupt neuronal proteostasis by sequestering proteasomes in a rare transition state, suggesting the cyclic conformational changes required for substrate processing (Collins & Goldberg, 2017) are altered by the association with the inclusions (Guo *et al*, 2018). Here, we aimed to elucidate gain‐of‐function mechanisms and the structure of cytoplasmic TDP‐43 aggregates found in sporadic and most genetic ALS/FTD cases. We focused on the aggregation‐prone TDP‐25 fragment (residues 220‐414 of full‐length human TDP‐43) (Neumann *et al*, 2006; Zhang *et al*, 2009) using a pipeline of cryo‐ET, proteomics, and functional assays. Neuronal TDP‐25 inclusions show amorphous morphology and gel‐like biophysical properties. The inclusions are enriched in stalled 26S proteasomes similar to poly‐GA inclusions, suggesting inhibition of the UPS is a common pathomechanism in ALS/FTD.

## Results and Discussion

### TDP‐25 inclusions are amorphous and enriched in 26S proteasomes

**Figure 1:**
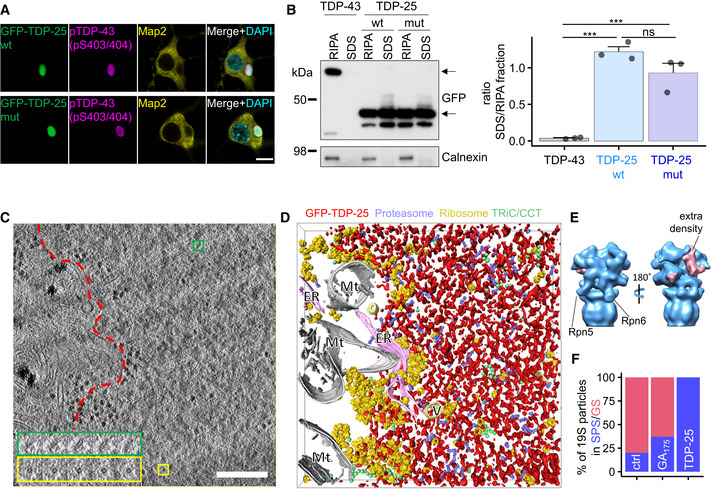
TDP‐25 forms amorphous inclusions enriched in proteasomes Primary rat hippocampal neurons were transduced with constructs encoding for GFP‐tagged TDP‐25 (amino acids 220‐414 of TDP‐43) on day 5 *in vitro* and cultured for 8 additional days (DIV5+8). Immunofluorescence of GFP‐TDP‐25 wild‐type and variant containing eight ALS‐causing mutations shows disease‐related phosphorylation at serines 403/404. Counterstain to label the neuronal cytoskeleton (MAP2) and nuclei (DAPI). Scale bar = 10 µm.Hippocampal neurons transduced with GFP‐TDP‐25 constructs (DIV5+8) or GFP‐TDP‐43 (DIV5+4 due to higher toxicity) were sequentially extracted with RIPA buffer, followed by 2% SDS buffer and analyzed via immuoblotting. Left panel shows representative immunoblot for GFP and loading control calnexin. Upper arrow marks GFP‐TDP‐43 band, lower arrow marks GFP‐TDP‐25 band. Right panel shows quantification of the ratio of the respective GFP densiometric signal in SDS extract to RIPA extract. Barplots showing means ± SD from *n* = 3 independent experiments. TDP‐43 (0.039 ± 0.029, mean ± CI) vs. TDP‐25 wt (1.22 ± 0.296) vs. TDP‐25 mut (0.928 ± 0.564): F(2,6) = 51.4, ***P = 0.000168, η² = 0.94. TDP‐43 vs. TDP‐25 wt: ***P = 1.67*10^−4^, TDP‐43 vs. TDP‐25 mut: ***P = 7.983*10^−4^, TDP‐25 wt vs. TDP‐25 mut: *P* = 0.1182, One‐way ANOVA with Tukey's *post‐hoc* test.Tomographic slice of an aggregate within a GFP‐TDP‐25 wild‐type transduced neuron (DIV5+8). Colored boxes show a series of higher magnification tomographic slices of representative protein complexes detected in the tomogram. Green boxes show side views of single‐capped, yellow boxes show ring‐like cross‐sections of 26S proteasomes. Red dotted line segments aggregate area. Scale bar = 200 nm.3D rendering of the aggregate shown in (C). Amorphous aggregate material is labeled in red, proteasomes in violet, ribosomes in yellow, TRiC/CCT chaperones in green, mitochondria (Mt) in white, endoplasmic reticulum (ER) in pink and other vesicles (V) in light yellow. The irregular aggregate structures were approximately segmented using a threshold‐based approach for visualization purposes. Average structures of proteasomes, ribosomes, and TRiC/CCT were pasted at the locations and orientations determined by template matching. See also Movie EV1.Subtomogram averaging of macromolecules in GFP‐TDP‐25 inclusions reveals the proteasome structure at ~20 Å resolution (see Fig EV1). The positions of Rpn5/PSMD11 and Rpn6/PSDM12 are indicated. Prominent extra densities in the substrate binding region are colored in pink in the 3D rendering.Classification based on the conformation of the regulatory particle in TDP‐25 inclusions compared with non‐transduced control neurons (Asano *et al*, 2015) and poly‐GA inclusions (Guo *et al*, 2018). GS: Ground State, SPS: Substrate Processing State. Due to the uncertainties inherent to the classification procedure, it is possible that a small fraction of particles adopted other conformations.

Expression of GFP‐tagged TDP‐25 in rat primary neurons resulted in abundant cytoplasmic inclusions phosphorylated at disease‐specific sites (Fig 1A) (Hasegawa *et al*, 2008). To explore the pathomechanisms enhanced by known pathogenic mutations, we additionally used a TDP‐25 variant containing eight mutations (G290A, G294V, G298S, A315T, M337V, G348C, N352S, and A382T) that individually cause ALS (Prasad *et al*, 2019). Wild‐type and mutant GFP‐TDP‐25 formed inclusions predominantly in the cell soma that were of similar appearance by light microscopy and showed a comparable degree of disease‐specific phosphorylation (TDP‐25 wild‐type: 95.8 ± 2.9% vs. TDP‐25 mutant: 96.2 ± 3.1%, mean ± SD from *n* = 3 biological replicates) (Fig 1A). While GFP‐TDP‐43 was almost completely soluble in RIPA buffer, a large fraction of wild‐type and mutant GFP‐TDP‐25 was only solubilized upon sequential extraction of the RIPA‐insoluble material with 2% SDS (Fig 1B) indicative of stronger intermolecular interactions in aggregating TDP‐25. Solubility of wild‐type and mutant TDP‐25 was not significantly different (Fig 1B).

**Figure EV1:**
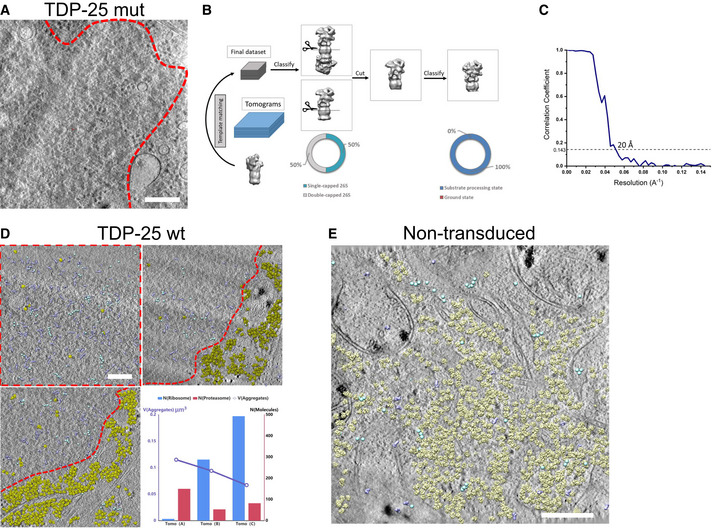
Proteasomes are enriched in TDP‐25 inclusions Tomographic slice of an aggregate within a neuron transduced with GFP‐TDP‐25 mutant construct (DIV5+8). Red dotted line segments aggregate area. Scale bar = 200 nm.Workflow of subtomogram averaging and classification. Subtomograms were identified using a low‐resolution single‐capped proteasome as template. All proteasomes were firstly classified into single‐capped or double‐capped. To further analyze the conformation of the regulatory particles, all proteasomes were cut *in silico* between the β‐rings of the core particle, resulting in two independent particles for double‐capped ones. 50% of the particles were assigned to the single and double‐capped classes, respectively. Cut out regulatory particles were merged and subjected to a further round of classification. All regulatory particles were assigned to substrate processing conformations. However, due to the uncertainties inherent to the classification procedure, it is possible that a small fraction of particles adopted other conformations.Gold‐standard Fourier shell correlation curve of the proteasome structure showing a resolution of 20 Å.Molecular mapping in three tomograms of GFP‐TDP‐25 wild‐type inclusions in transduced neurons (DIV5+8). Regions containing GFP‐TDP‐25 are outlined in red. For the whole tomogram, proteasomes (purple), TRiC (cyan) and ribosomes (yellow) are mapped to their original positions and orientations using the information from template matching and subtomogram averaging. The numbers of proteasomes and ribosomes detected in the tomograms are plotted versus the volume of the tomogram. Scale bar = 200 nm.Molecular mapping in a tomogram recorded on a non‐transduced control neuron, with proteasomes (purple), ribosomes (yellow) and TRiC (cyan) plotted as in Fig EV1D. This tomogram contains 24 proteasomes, 1,222 ribosomes and 73 TRiC molecules. Scale bar = 200 nm.

To investigate the downstream consequences of TDP‐25 aggregation, we analyzed neuronal GFP‐TDP‐25 inclusions *in situ* using cryo‐ET (Bauerlein *et al*, 2020). To that end, neurons were cultured, transduced with GFP‐tagged TDP‐25 constructs (wild‐type and mutant) and vitrified on EM grids. Vitrified grids were imaged by cryo‐light microscopy to determine the subcellular location of TDP‐25 inclusions. Correlative light‐electron microscopy (CLEM) was performed to locate back the inclusions in a dual‐beam cryo‐focused ion beam/scanning electron microscope (cryo‐FIB/SEM). Thin (100–200 nm) lamellae were prepared at those positions. Finally, lamellae were transferred to a cryo‐transmission electron microscope, where they were imaged at high magnification. Wild‐type TDP‐25 inclusions appeared amorphous and seemingly lacked fibrillar structure, although they were clearly demarcated within the cytoplasm (Fig 1C and D, Movie EV1). These findings are in stark contrast to previously investigated poly‐Q, poly‐GA, and α‐synuclein neuronal inclusions, which show amyloid‐like conformation in neurons in our cryo‐ET pipeline (Bauerlein *et al*, 2017; Guo *et al*, 2018; Trinkaus *et al*, 2021). Introducing the eight ALS‐causing mutations had no obvious effect on the morphology of TDP‐25 inclusion (Fig EV1A).

Similar to our findings in *C9orf72* poly‐GA inclusions (Guo *et al*, 2018), we detected ring‐like structures (Fig 1C) accumulating within TDP‐25 inclusions. Sub‐tomogram averaging of these objects (Fig EV1B) converged to a proteasome structure at ~20 Å resolution (Fig 1E, Fig EV1C). Interestingly, an extra density that was not accounted for by proteasomal subunits was present on the proteasome regulatory particle, possibly reflecting substrates or adaptor proteins (Fig 1E).

Whereas ribosomes were largely excluded from TDP‐25 inclusions, we found an approximately eightfold enrichment of proteasomes compared with proteasome concentration in control neurons (Asano *et al*, 2015; Guo *et al*, 2018; Figs 1D and EV1D and E). Strikingly, classification based on the conformation of 19S regulatory particles revealed that virtually all proteasome particles within the inclusions were in substrate processing states (Fig 1E and F). In comparison, only 20 and 37% of proteasomes were in substrate processing states in control neurons and poly‐GA inclusions, respectively (Asano *et al*, 2015; Guo *et al*, 2018; Fig 1F). It should be noted that, due to the uncertainties inherent to the classification procedure, we cannot rule out that a small fraction of particles adopted other conformations. The little to no detectable amount of ground state proteasomes suggests that proteasomes inside TDP‐25 inclusions are stalled, as proteasome function requires cyclic transition through activated and ground states (Collins & Goldberg, 2017).

### Amorphous TDP‐25 inclusions have gel‐like properties

**Figure 2:**
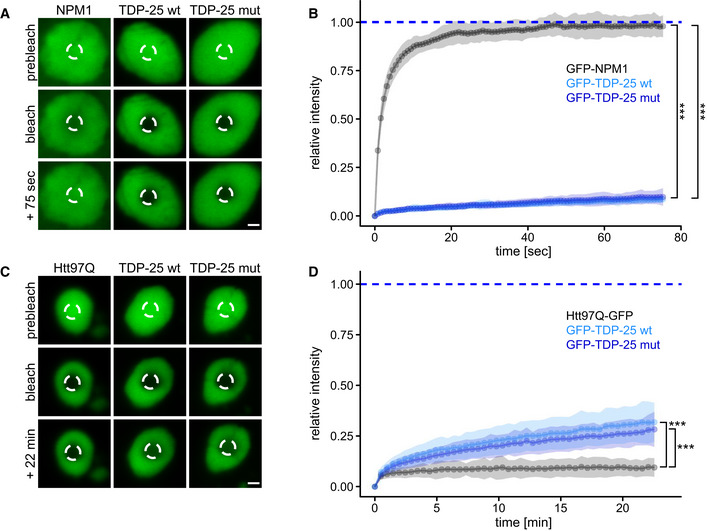
TDP‐25 inclusions are neither liquid‐like nor solid‐like A–DPrimary rat hippocampal neurons were transduced with GFP‐TDP‐25 variants, GFP‐NPM1 or Htt97Q‐GFP and analyzed by fluorescence recovery after photobleaching (FRAP) at DIV5+8, except Htt97Q‐GFP which was analyzed at DIV5+6 to avoid excessive toxicity. Example images with indicated bleach regions (dashed circles) are shown (A, C). Scale bar = 1.5 µm. Resulting normalized FRAP curves (relative fluorescence intensity over time) with values representing means ± SD are shown in (B, D). (B) Comparison of the averaged recovery fractions of the last six timepoints obtained from three independent experiments. GFP‐NPM1 (0.982 ± 0.019, mean ± CI, *n* = 37 cells) vs. GFP‐TDP‐25 wt (0.097 ± 0.013, *n* = 35 cells) vs. GFP‐TDP‐25 mut (0.085 ± 0.006, *n* = 38 cells): H(1) = 74.134, *df* = 2, ***P < 2.2*10^−16^, η²[H] = 0.674, Kruskal‐Wallis Test. GFP‐NPM1 vs. GFP‐TDP‐25 wt: ***P < 2*10^−16^, GFP‐NPM1 vs. GFP‐TDP‐25 mut: ***P < 2*10^−16^, GFP‐TDP‐25 wt vs. GFP‐TDP‐25 mut: *P* = 0.11, Pairwise Wilcoxon Rank Sum Tests with Benjamini‐Hochberg correction. In (D), the recovery fractions averaged at >20 min timepoints were compared from four independent experiments. Htt97Q‐GFP (0.094 ± 0.017, *n* = 25 cells) vs. GFP‐TDP‐25 wt (0.312 ± 0.041, *n* = 23 cells) vs. GFP‐TDP‐25 mut (0.271 ± 0.029, *n* = 24 cells): H(1) = 46.376, *df* = 2, ***P = 8.501*10^−11^, η²[H] = 0.643, Kruskal‐Wallis Test. Htt97Q‐GFP vs. GFP‐TDP‐25 wt: ***P = 1.9*10^−11^, Htt97Q‐GFP vs. GFP‐TDP‐25 mut: ***P = 4.3*10^−12^, GFP‐TDP‐25 wt vs. GFP‐TDP‐25 mut: *P* = 0.12, Pairwise Wilcoxon Rank Sum Tests with Benjamini‐Hochberg correction.

**Figure EV2:**
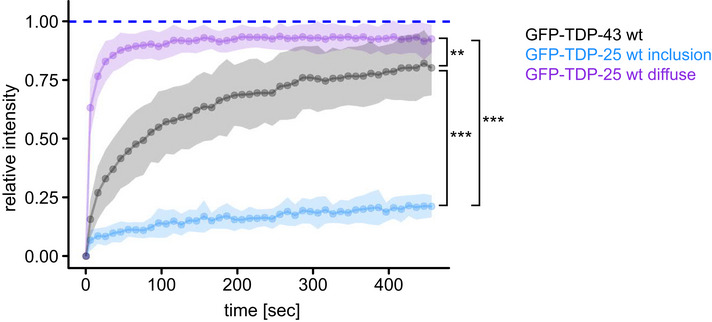
TDP‐25 inclusions are less mobile than TDP‐43 Rat primary hippocampal neurons were transduced with GFP‐TDP‐25 wild‐type (DIV5+8) or GFP‐TDP‐43 wild‐type (DIV9+4) and analyzed by FRAP. The resulting normalized FRAP curves (relative fluorescence intensity over time) with values representing means ± SD are shown. Comparison of the averaged recovery fractions of the last six timepoints obtained from three independent experiments: GFP‐TDP‐43 (0.802 ± 0.06, mean ± CI, *n* = 20 cells) vs. GFP‐TDP‐25 inclusion (0.209 ± 0.019, *n* = 18 cells) vs. GFP‐TDP‐25 diffuse (0.926 ± 0.045, *n* = 10 cells): H(1) = 36.295, *df* = 2, *P* = 1.314*10^−8^, η²[H] = 0.762, Kruskal‐Wallis Test. GFP‐TDP‐43 vs. GFP‐TDP‐25 inclusion: ***P = 1.8*10^−10^, GFP‐TDP‐43 vs. GFP‐TDP‐25 diffuse: **P = 0.0033, GFP‐TDP‐25 inclusion vs. GFP‐TDP‐25 diffuse: ***P = 2.3*10^−7^, Pairwise Wilcoxon Rank Sum Tests with Benjamini‐Hochberg correction.

Given the amorphous appearance of TDP‐25 inclusions, we asked whether they may represent phase‐separated liquid droplets. Thus, we analyzed the mobility of GFP‐TDP‐25 in neuronal inclusions using fluorescence recovery after photobleaching (FRAP) in comparison with known liquid and solid reference proteins, that is, nucleolar NPM1 and inclusion‐forming poly‐Q, respectively (Bauerlein *et al*, 2017; Frottin *et al*, 2019). In contrast to TDP‐25, GFP‐NPM1 fluorescence recovered within seconds after bleaching, consistent with high mobility of the protein within the liquid‐like nucleolus (Fig 2A and B). This clearly argues against a liquid droplet character of TDP‐25 inclusions. Some neurons showed diffuse cytoplasmic TDP‐25 expression without inclusion formation. Photobleaching of such diffuse TDP‐25 resulted in very quick recovery suggesting that inclusion formation greatly reduces mobility (Fig EV2). Moreover, nuclear full‐length TDP‐43 showed a much higher mobile fraction than TDP‐25 inclusions (Fig EV2), in line with its good solubility (Fig 1B). However, compared with fibrillar poly‐Q (huntingtin exon 1 containing 97 glutamines fused to GFP; Htt97Q‐GFP), a classical amyloid previously shown to interact with ER membranes (Bauerlein *et al*, 2020), TDP‐25 mobility was much higher (~10% vs. ~25% recovery at 22 min, Fig 2C and D).

Overall, these findings suggest that non‐fibrillar TDP‐25 adopts a specific conformation within the inclusions that is best described by a gel‐like state. Solidification into hydrogels has been reported for phase‐separated droplets of FUS, involving the formation of amyloid‐like fibrils (Murray *et al*, 2017; Qamar *et al*, 2018). A similar process has been postulated for TDP‐43, and very recently, amyloid‐like fibrils of TDP‐43 have been obtained from ALS/FTD patient brains (Guenther *et al*, 2018; Babinchak *et al*, 2019; Cao *et al*, 2019; Gasset‐Rosa *et al*, 2019; Mann *et al*, 2019; Li *et al*, 2021; Arseni *et al*, 2022). Taken together, GFP‐TDP‐25 inclusions are clearly less dynamic than classical phase‐separated compartments, but more dynamic than fibrillar aggregates.

### TDP‐25 sequesters and impairs the proteasome

**Figure 3:**
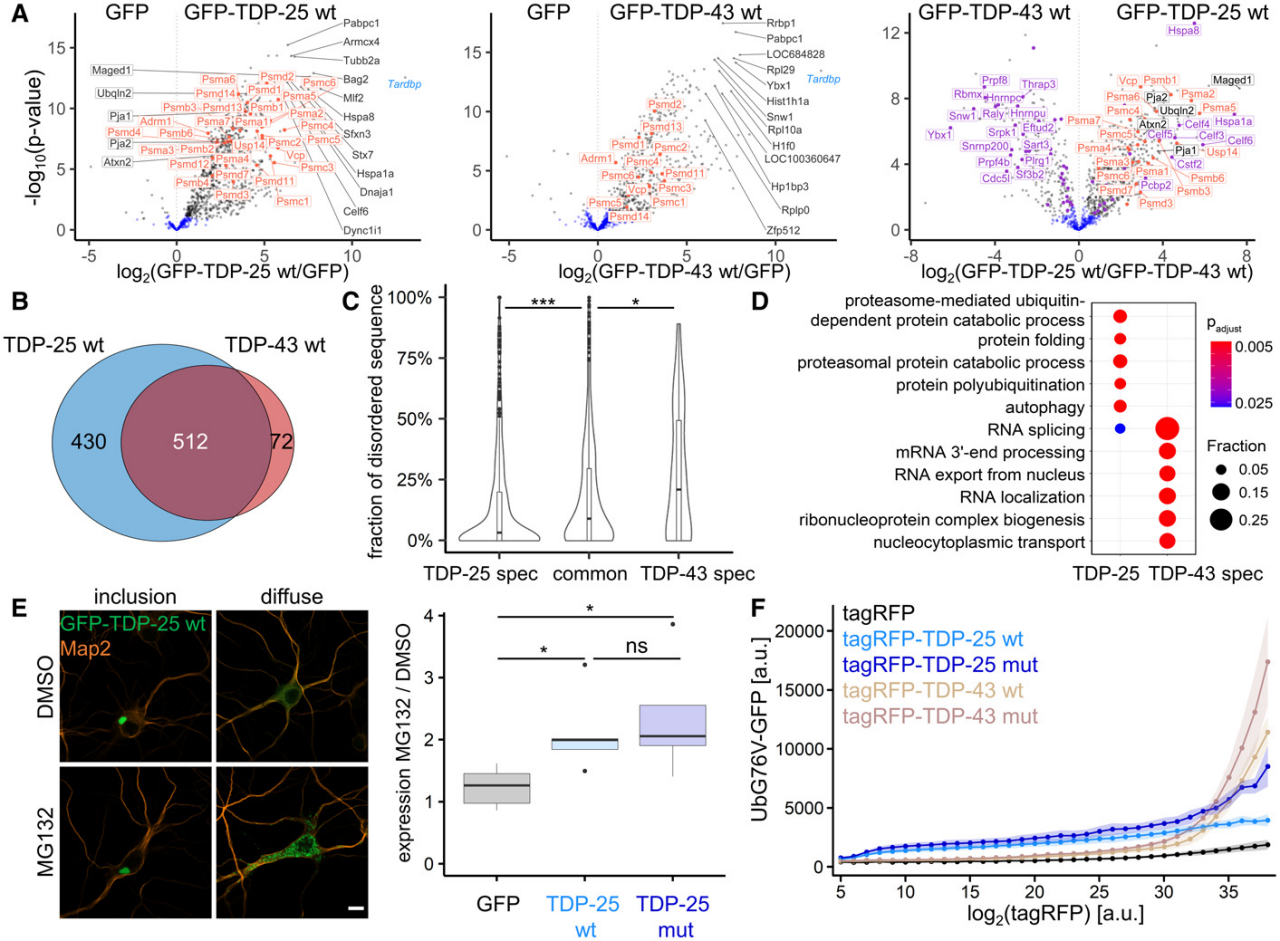
TDP‐25 sequesters and impairs the proteasome Rat primary cortical neurons were transduced with GFP‐TDP‐25 wild‐type, GFP‐TDP‐43 wild‐type or GFP at DIV5. Due to high toxicity, GFP‐TDP‐43 (*n* = 6) and GFP control samples (*n* = 6) were harvested at DIV5+4, while GFP‐TDP‐25 (*n* = 5) and additional GFP control samples (*n* = 6) were harvested at DIV5+8. Immunoprecipitates were analyzed by LC‐MS/MS. Volcano plots indicate enrichment and statistical significance (gray dots: FDR‐corrected *P* < 0.05). Proteins associated with GO term “proteasome complex” (GO:0000502) are labeled in orange and proteins associated with GO term “RNA splicing” (GO:0008380) in purple. Full data are listed in Dataset EV1. A comparison with previous proteomic studies is shown in Fig EV3A.Overlap of GFP‐TDP‐25 and GFP‐TDP‐43 interactomes. Significant interactors in FDR‐based approach (see Methods) were considered for comparison.Combined violin and box plot (box shows lower quartile, median and upper quartile, whiskers extend to the most extreme data point within 1.5 the interquartile range of the box) of the proportion of low‐complexity regions (D2P2) of wild‐type GFP‐TDP‐25‐specific, GFP‐TDP‐43‐specific or shared/common interactors. TDP‐25‐specific (0.144 ± 0.021, *n* = 432 Uniprot IDs) vs. TDP‐43‐specific (0.266 ± 0.061, *n* = 78) vs. common interactors (0.184 ± 0.02, *n* = 481): H(1) = 22.276, *df* = 2, *P* = 1.455*10^−5^, η²[H] = 0.0205, Kruskal‐Wallis test. TDP‐25‐specific vs. TDP‐43‐specific: ***P = 0.00054, TDP‐25‐specific vs. common: ***P = 0.00035, TDP‐43‐specific vs. common interactors: *P = 0.04893, Pairwise Wilcoxon Rank Sum Tests with Benjamini‐Hochberg correction.Gene ontology (GO) enrichment analysis (biological process) in interactors specific for either wild‐type GFP‐TDP‐25 or GFP‐TDP‐43. Full list of GO terms is found in Dataset EV2.Immunofluorescence of hippocampal neurons transduced with GFP, GFP‐TDP‐25 wild‐type or mutant and treated with MG132 (10 µM, 14 h, DIV5+8). Representative images of GFP‐TDP‐25 inclusions and diffuse cytoplasmic staining. Automated quantification of GFP intensity normalized to the number of DAPI‐stained nuclei (omitted here for clarity) in MG132‐treated cells compared to DMSO control. Counterstain to label the neuronal cytoskeleton (MAP2). Scale bar = 10 µm. Box plot as in Fig 3C. GFP (1.23 ± 0.393, *n* = 5 independent experiments) vs. GFP‐TDP‐25 wt (2.11 ± 0.804, *n* = 5) vs. GFP‐TDP‐25 mut (2.36 ± 1.16, *n* = 5): H(1) = 7.34, *df* = 2, *P* = 0.02548, η²[H] = 0.445, Kruskal‐Wallis test. GFP vs. GFP‐TDP‐25 wt: *P = 0.048, GFP vs. GFP‐TDP‐25 mut: *P = 0.048, GFP‐TDP‐25 wt vs. GFP‐TDP‐25 mut: *P* = 0.690, Pairwise Wilcoxon Rank Sum Tests with Benjamini‐Hochberg correction.A HEK293 cell line expressing the proteostasis reporter UbG76V‐GFP was transiently transfected with tagRFP, tagRFP‐TDP‐25 wild‐type, tagRFP‐TDP‐25 mutant, tagRFP‐TDP‐43 wild‐type or tagRFP‐TDP‐43 mutant. UbG76V‐GFP levels were analyzed by flow cytometry 72 h after transfection. The relationship of binned tagRFP fluorescence and UbG76V‐GFP fluorescence is plotted. Values shown are means ± SD obtained from *n* = 4 independent experiments. For clarity, adjusted *P*‐values of pairwise comparisons within each tagRFP intensity bin are shown in Fig EV3C. Note that at lower expression levels (bin < 30) both TDP‐25 variants induce higher UbG76V‐GFP levels than their respective full‐length counterparts, indicative of stronger impairment of proteostasis. The full‐length variants show meaningful accumulation of the reporter levels only at the high‐expression levels. The mutant versions of TDP‐25 and TDP‐43 lead to an increased reporter signal at higher expression levels compared with wild‐type variants.

**Figure EV3:**
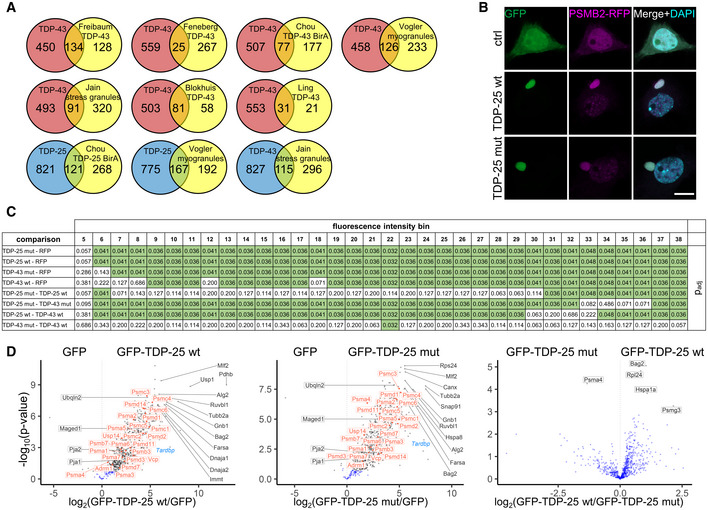
Additional interactome analyses Proteins identified in the GFP‐TDP‐43 interactome from Fig 3A (uniquely or in a cluster) were compared to published datasets (Freibaum *et al*, 2010; Ling *et al*, 2010; Blokhuis *et al*, 2016; Chou *et al*, 2018; Feneberg *et al*, 2020). LOC and RGD gene names that usually represent minor components of the identified clusters were removed, because such entries could not be mapped based on gene names between species. A core set of 32 proteins from our TDP‐43 interactome was found in four (DDX3X, DHX9, ELAVL1, HNRNPA0, HNRNPD, HNRNPH1, HNRNPL, HNRNPM, HNRNPR, ILF2, MATR3, PABPC4, RALY, YBX1) or three (DDX5, DDX6, EFTUD2, EIF3A, HNRNPA3, HNRNPAB, HNRNPC, HNRNPK, HNRNPU, HNRNPUL2, ILF3, NONO, NOP56, NOP58, PABPC1, SNRNP200, SNRPA1, SSB) interactome datasets (Freibaum *et al*, 2010; Ling *et al*, 2010; Blokhuis *et al*, 2016; Feneberg *et al*, 2020). There was also substantial overlap of our TDP‐25 interactome with a BirA proximity labeling dataset for TDP‐25 (Chou *et al*, 2018), the stress granule proteome (Jain *et al*, 2016) and TDP‐43 myogranules in regenerating muscle (Vogler *et al*, 2018).Colocalization of proteasomes with TDP‐25 inclusions. Primary rat hippocampal neurons were co‐transduced with lentivirus encoding for GFP‐tagged TDP‐25 variants or GFP and PSMB2‐tagRFP lentivirus on day 5. Immunofluorescence images were taken 8 days after transduction (DIV5+8). Counterstain to label nuclei (DAPI). Scale bar = 10 µm.Statistical analysis for Fig 3F. Pairwise comparisons using Wilcoxon Rank Sum Tests with Benjamini‐Hochberg correction were performed for each tagRFP intensity bin respectively. Comparisons with *P* < 0.05 are highlighted in green.Interactome analysis of TDP‐25 mutant. Rat primary cortical neurons were transduced with GFP‐TDP‐25 wild‐type (*n* = 5), GFP‐TDP‐25 mutant (*n* = 5) or GFP (*n* = 4) at DIV5 and harvested at DIV5+8. Immunoprecipitates were analyzed by LC‐MS/MS. Volcano plots indicate enrichment and statistical significance (gray dots: FDR‐corrected *P* < 0.05). Proteins associated with GO term “proteasome complex” (GO:0000502) are labeled in orange. Full data are listed in Dataset EV3. This data was obtained from a second experiment independent from Fig 2A.

Phase‐separated inclusions may form through weak multivalent interactions with other cellular components (Martin & Mittag, 2018; Gomes & Shorter, 2019). To further investigate this gel‐like state and the molecular consequences of the TDP‐25 inclusions, we analyzed the interactomes of TDP‐25 and full‐length TDP‐43 using mass‐spectrometry‐based proteomics. We detected several hundred specific interactors for full‐length TDP‐43 and TDP‐25 in primary neurons, in comparison with the GFP‐only control (Figs 3A and B, and EV3A, Dataset EV1). TDP‐43‐specific interactors were strongly enriched in splicing‐associated proteins consistent with the prominent involvement of TDP‐43 in RNA splicing and in line with previous interactome studies for TDP‐43 (Figs 3A and D, and EV3A; Prasad *et al*, 2019).

The loss of the N‐terminus in TDP‐25 led to the loss of 72 interactors compared with full‐length TDP‐43 but resulted in over 400 new interactions, which may shape biophysical properties and drive gain‐of‐function toxicity in ALS/FTD (Fig 3B). This gain of interactors may be explained by misfolding of the protein fragment (Gottlieb *et al*, 2019) or co‐partitioning of additional proteins in the gel‐phase and is consistent with previous findings using proximity labeling (Chou *et al*, 2018). To investigate the role of LLPS in inclusion formation in our neuronal model, we analyzed the content of low‐complexity domains or intrinsically disordered regions in interacting proteins, as such sequences drive phase separation through multivalent homo‐ or heterotypic interactions (Martin & Mittag, 2018). Interestingly, the proportion of disordered regions/low‐complexity regions was lower in TDP‐25‐specific interactors than in TDP‐43‐specific and shared interactors (Fig 3C), in line with the higher dynamics of nuclear TDP‐43 (Fig EV2). The relative absence of proteins with low‐complexity domains within TDP‐25 inclusions argues against their formation through LLPS or may reflect solidification driven by loss of LLPS‐promoting low‐complexity interactors.

Consistent with our cryo‐ET data, proteomics showed that a large number of proteasome subunits were highly enriched in the TDP‐25 interactome (Fig 3A and D and Dataset EV2). Furthermore, immunofluorescence confirmed partial sequestration of proteasomes into GFP‐TDP‐25 inclusions (Fig EV3B), similar to poly‐GA inclusions in *C9orf72* ALS/FTD (Guo *et al*, 2018), although proteasomal sequestration was less prominent for TDP‐25. Our mass spectrometry data also confirmed the previously reported selective interaction of TDP‐25 with other UPS components, such as the ALS‐linked Ubiquilin 2 (Deng *et al*, 2011; Cassel & Reitz, 2013) and the E3 ubiquitin ligases Pja1 (Watabe *et al*, 2020), which could indicate ongoing degradation attempts. These or other UBL domain proteins may contribute to the extra density found on the proteasome regulatory particle (Fig 1E).

Finally, we investigated the functional consequences of proteasome recruitment to TDP‐25 inclusions and asked whether TDP‐25 is primarily a substrate or an inhibitor of the proteasome. Treating GFP‐TDP‐25 expressing neurons with the proteasome inhibitor MG132 increased the level of punctate GFP‐TDP‐25, without obvious effects on the larger inclusions (Fig 3E), in line with a previous report (Scotter *et al*, 2014). Quantification showed a similar twofold accumulation of GFP‐TDP‐25 wild‐type and mutant upon proteasome inhibition, suggesting that the proteasome is actively degrading largely soluble GFP‐TDP‐25 under basal conditions.

Our cryo‐ET data suggested alterations in the functional cycle of proteasomes recruited to TDP‐25 inclusions. To interrogate whether TDP‐25 affects the cellular proteasome capacity, we expressed RFP‐TDP‐25 in an established UbG76V‐GFP reporter HEK293 cell line (Dantuma *et al*, 2000). Already starting at low expression levels, we noticed a robust dose‐dependent accumulation of the UPS reporter in cells expressing wild‐type and mutant RFP‐TDP‐25 compared with the RFP‐only control and full‐length RFP‐TDP‐43, indicative of competitive inhibition (Fig 3F). Compared with RFP‐only control, RFP‐TDP‐43 induced strong upregulation of the reporter only at higher expression levels, which is consistent with an overall less pronounced interaction of TDP‐43 with the proteasome subunits compared with TDP‐25 (Fig 3A). Introducing the eight ALS‐causing mutations into TDP‐25 and TDP‐43 further impaired overall protein degradation at high‐expression levels, although this only reached statistical significance for TDP‐25 (Figs 3F and EV3C). Nevertheless, the interactomes of wild‐type and mutant TDP‐25 appeared very similar (Fig EV3D). These findings corroborate our structural data, indicating that proteasomes enriched in TDP‐25 inclusions are functionally impaired similar to proteasomes sequestered in poly‐GA inclusions (Guo *et al*, 2018).

In summary, we used cryo‐ET, proteomics and functional assays in primary neurons to show that TDP‐43 C‐terminal fragments adopt a gel‐like conformation and impair proteostasis by sequestering stalled proteasomes *in situ*. This gel‐like conformation could be a transition state from a liquid compartment (Conicella *et al*, 2016; Yu *et al*, 2021) to mature amyloid fibrils (Arseni *et al*, 2022) or an alternative state like the SDS‐resistant cytoplasmic myo‐granules of full‐length TDP‐43 found in regenerating muscle that lack classical amyloid conformation (Vogler *et al*, 2018). So far, cryo‐ET has only detected proteasome accumulation and impairment within inclusions related to ALS/FTD (Guo *et al*, 2018 and this article), but not in other protein aggregates such as poly‐Q, artificial β‐sheet proteins or α‐synuclein (Bauerlein *et al*, 2017; Riera‐Tur *et al*, 2021; Trinkaus *et al*, 2021). Additionally, we show that ALS‐causing mutations may further impair proteasome function. These data resonate with previous reports on the crucial role of the UPS for motor neuron function and survival (Bett *et al*, 2009; Tashiro *et al*, 2012; Bax *et al*, 2019), pointing to proteasome dysfunction as a major specific hallmark of ALS/FTD pathogenesis.

## Materials and Methods

### Primary rat neurons

Hippocampi and neocortices from E19 rat embryos were dissected in ice‐cold dissection media (HBBS, 1% penicillin/streptomycin, 10 mM HEPES pH 7.3, all Thermo Fisher Scientific), followed by enzymatic dissociation in dissection media (15 min in 0.15% trypsin for hippocampi, 20 min in 0.25% trypsin, 0.7 mg/ml DNase I for cortices). Hippocampal neurons were plated in Neurobasal media supplemented with 2% B27, 1% penicillin‐streptomycin, 0.5 mM L‐glutamine and 12.5 µM L‐glutamate (all Thermo Fisher Scientific) at 85,000 cells/ml on gold grids for cryo‐ET experiments, on poly‐D‐lysine‐coated glass coverslips (VWR) in 12‐well plates for immunofluorescence, in 35 mm petri dishes with a 14 mm glass bottom inlay (MatTek Corporation) for FRAP analysis and in 6‐well plates for biochemical analysis. For mass spectrometry, cortical neurons were plated in 10 cm petri dishes at a density of 400,000 cells/ml in the above media without L‐glutamate supplementation.

### DNA constructs

cDNA encoding for TDP‐25 (amino acids 220‐414), TDP‐43 (amino acids 1‐414) and NPM1 were PCR amplified from HEK293FT (Thermo Fisher Scientific) cDNA and subsequently cloned into the FhSynW backbone (human synapsin promoter) with an N‐terminal EGFP tag (May *et al*, 2014). TDP‐43 variants with N‐terminal tagRFP were cloned in a lentiviral backbone with UbC promoter (FUW3), while human PSMB2 was expressed with a C‐terminal tagRFP. TDP‐43 point‐mutations were introduced using standard PCR methods. The Htt97Q‐EGFP construct was generated by subcloning the open reading frame from Addgene #1186 into FhSynW.

### Lentiviral packaging and transduction

HEK293FT cells of low passage number were seeded into three 10 cm dishes (5 × 10^6^ cells/dish) using DMEM (Thermo Fisher Scientific) supplemented with 10% fetal bovine serum (Sigma‐Aldrich), 1% penicillin‐streptomycin and 1% Non‐Essential Amino Acids (both Thermo Fisher Scientific) as described previously (Guo *et al*, 2018). On the next day, cells were co‐transfected with 18.6 µg transfer vector, 11 µg pSPAX2 and 6.4 µg pVSVG using Lipofectamine 2000 (Thermo Fisher Scientific). The transfection media was replaced by plating media supplemented with 13 mg/ml bovine serum albumin (Sigma Aldrich) on the following day. Lentivirus from the cell supernatant was collected 24 h later by ultracentrifugation (87,000 *g*, 2 h, 4°C). Finally, lentiviral particles were resuspended in Neurobasal media and stored at −80°C in aliquots.

### Immunofluorescence, image acquisition and quantitative analysis

Cells grown on coverslips were briefly washed with PBS once before fixing for 10 min at room temperature using 4% paraformaldehyde (Sigma‐Aldrich) and 4% sucrose (Sigma‐Aldrich) in PBS. Primary antibodies (anti‐phospho TDP‐43 [pS403/404], Cat# TIP‐PTD‐P05, Cosmo Bio Co., Ltd., 1:1,000; anti‐Map2, Cat# M1406, clone AP‐20, Sigma‐Aldrich, 1:250) as well as secondary antibodies (Goat anti‐rabbit Alexa 555 or Goat anti‐mouse Alexa 647, Thermo Fisher Scientific, 1:400) were diluted in GDB buffer containing 0.1% gelatin (Sigma‐Aldrich), 0.3% Triton X‐100 (Merck), 450 mM NaCl, and 16 mM sodium phosphate pH 7.4. Coverslips were incubated with primary antibodies overnight at 4°C and secondary antibodies for 1 h at room temperature, each followed by three washes with PBS. Coverslips were mounted using Vectashield Vibrance with DAPI (Cat# VEC‐H‐1800, Biozol) to counterstain nuclei.

For colocalization studies, confocal microscopy was performed using an inverted Zeiss LSM800 Axio Observer.Z1 / 7 confocal laser scanning microscope (Carl Zeiss) with a Plan‐Apochromat 63×/1.40 Oil DIC M27 objective and equipped with the ZEN 2.5 software package (blue edition, Carl Zeiss). All images were acquired at 2048 × 2048 pixel resolution with two‐times averaging using two GaAsP PMT detectors at 8‐bit depth.

To quantify effects of proteasomal inhibition (MG132 vs. DMSO), tile scan images (> 100 tiles per condition) were taken on a Leica DMi8 fluorescence microscope using a HC PL APO 40×/0,95 CORR objective, a DFC9000 camera and LAS X Software (Leica Microsystems). Images were quantified via ImageJ/Fiji software (version 1.53c) to determine differences in GFP signal between conditions using a custom script. Integrated Density of GFP and GFP‐TDP‐25 variants were measured using a fixed threshold, and the resulting medians were normalized to the number of detected nuclei. DAPI‐stained nuclei were counted as particles with circularity factor 0.5–1.0 and size between 60 and 150 µm^2^ after thresholding and binary water shedding.

### Cellular fractionation and immunoblotting

Transduced neurons were washed with PBS and lysed in RIPA buffer (137 mM NaCl, 20 mM Tris‐HCl pH 7.5, 0.1% SDS, 10% glycerol, 1% Triton X‐100, 0.5% deoxycholate, 2 mM EDTA) freshly supplemented with 67 U/ml Benzonase Nuclease (Sigma‐Aldrich), protease inhibitor cocktail (Sigma‐Aldrich) and phosphatase inhibitor cocktail (Sigma‐Aldrich) on ice for 30 min. After centrifugation (18,000 *g*, 30 min, 4°C), the supernatant was collected as RIPA‐soluble fraction. Pellets were resuspended in 2% SDS buffer (2% SDS, 100 mM Tris‐HCl pH 7.0) and incubated for 2 h at room temperature. Upon centrifugation (18,000 *g*, 30 min, 4°C), the supernatant was collected as SDS‐soluble fraction. Samples were denatured for 10 min at 95°C after adding 3× loading buffer (200 mM Tris‐HCl pH 6.8, 6% SDS, 20% glycerol, 0.1 g/ml DTT, 0.1 mg Bromophenol Blue). Protein concentrations were determined for the RIPA‐soluble fraction by BCA assay (Interchim) and equivalent fractions of the total cell population were loaded on 10% SDS–PAGE gels, followed by transfer to Immobilon^®^‐P PVDF membranes (Merck). Membranes were blocked in 0.2% iBlock (Thermo Fisher Scientific). The following primary antibodies were used: anti‐GFP (Cat# 75‐131, clone N86/8, UC Davis/NIH Neuromab Facility) and anti‐Calnexin (Cat# ADI‐SPA‐860, Enzo Life Sciences).

### CLEM, cryo‐FIB and cryo‐ET

CLEM, cryo‐FIB/SEM and cryo‐ET tomographic data collection was performed as described in detail before (Guo *et al*, 2018). In brief, EM grids were mounted onto modified Autogrids sample carriers (Rigort *et al*, 2012) and then transferred into the cryo‐stage of an FEI CorrSight microscope. Images of grid and GFP signal were, respectively, acquired with FEI MAPS software in transmitted light and widefield mode using 5× and 20× lenses. The samples were then transferred into a FIB/SEM dual‐beam microscope (Quanta 3D FEG, FEI) using a cryo‐transfer system (PP3000T, Quorum). Cryo‐light microscope and SEM images were correlated in 2D with MAPS 2.1 software (Thermo Fisher Scientific). Lamellas were prepared using Ga^2+^ ion beam at 30 kV in the regions of GFP signal with final thickness of 100–200 nm.

The grids were transferred to an FEI Titan Krios transmission electron microscope for tomographic data collection. For the whole procedure, samples were kept at liquid N_2_ temperature. Tomographic tilt series were recorded with a Gatan K2 Summit direct detector in counting mode. A GIF‐quantum energy filter was used with a slit width of 20 eV to remove inelastically scattered electrons. Tilt series were collected dose‐symmetrically between −51° and +69° starting from +12° with an increment of 3° and total dose of 110 e^−^/Å^2^ using SerialEM software (Mastronarde, 2005) at a pixel size of 3.52 Å.

### cET Image processing

Image frames were aligned using Motioncor2 (Zheng *et al*, 2017). IMOD software package (Kremer *et al*, 1996) was used for tomogram reconstruction: The tilt series were firstly aligned using fiducial‐less patch tracking, and tomograms were then reconstructed by weighted back projection of the resulting aligned images. Contrast was enhanced by filtering the tomograms using MATLAB script tom_decov (https://github.com/dtegunov/tom_deconv).

MATLAB with TOM toolbox (Nickell *et al*, 2005) was used as general platform for image processing. For segmentation, tomograms were rescaled with a binning factor of four. The membranes were firstly segmented automatically with TomoSegMemTV (Martinez‐Sanchez *et al*, 2014) using a tensor voting method, and then manually optimized with Amira (Thermo Fisher Scientific). Proteasome, ribosome, and TRiC were detected using an template matching procedure with PyTOM software (Hrabe *et al*, 2012), templates were generated by filtering the corresponding structures from previous work (Guo *et al*, 2018) to 40 Å. The resulting coordinates were used to crop the full size subtomograms from the original tomograms, which were then CTF corrected, classified and refined using RELION (Bharat & Scheres, 2016). In total, 1,170 proteasome subtomograms were picked from 11 tomograms with dominant TDP‐25 aggregates for further analysis.

### FRAP assay

FRAP (fluorescence recovery after photobleaching) experiments were performed on transduced hippocampal neurons on glass bottom dishes at 37°C in HBSS buffer (Thermo Fisher Scientific) supplemented with 20 mM HEPES (Thermo Fisher Scientific) and 4.5 g/l glucose using an inverted LSM710 Axio Observer.Z1 confocal laser scanning system (Carl Zeiss) equipped with a Plan‐Apochromat 63×/1.40 Oil DIC M27 objective, a PMT detector and the ZEN 2011 software (black edition, Carl Zeiss). Images were acquired with two‐line averages. Three prebleach images were taken. Within the investigated structure (inclusion or nucleolus), a circular region of interest (ROI) of approx. 1.3 µM diameter was photobleached at 100% laser power and fluorescent recovery was monitored over 75 s (image acquisition in 0.8 s intervals), 22 min (30 s intervals), or 450 s (10 s intervals). For analysis, recorded movies were aligned using the *StackReg* plugin of the ImageJ/Fiji software. Afterwards, the mean intensity values of each region of interest (ROI) were monitored over time with ROI1 being the bleached region, ROI2 the whole nucleolus/inclusion/nucleus/surrounding cytoplasm and ROI3 the background noise. Values were then analyzed using easyFRAP (version 9.0.1) by applying the “full scale” normalization method (Rapsomaniki *et al*, 2012).

### Immunoprecipitation

Transduced neurons were lysed in 2% Triton X‐100, 750 mM NaCl, 1 mM KH_2_PO_4_, freshly supplemented with 67 U/ml Benzonase Nuclease, 1× protease inhibitor cocktail and 1× phosphatase inhibitor cocktail under constant rotation for 45 min at 4°C and then centrifuged at low speed (1,000 *g*, 5 min, 4°C) to remove cellular debris but retain inclusion proteins (Hartmann *et al*, 2018). Supernatants were incubated with anti‐GFP antibody (Cat# N86/38, clone N86/38, UC Davis/NIH NeuroMab) pre‐bound to Protein G Dynabeads (Cat# 10004D, Life Technologies) on a shaking rotator for 3 h at 4°C. Beads were subsequently washed three times (150 mM NaCl, 50 mM Tris‐HCl pH 7.5, 5% glycerol) and analyzed by mass spectrometry.

### Sample preparation for LC‐MS/MS

Beads were resuspended in 155 µl denaturing buffer (8 M urea, 50 mM Tris‐HCl pH 7.5, 1 mM dithiothreitol). Proteins were digested off the beads with LysC (220 ng/sample) for 1 h at room temperature while shaking at 1,200 rpm. The suspension was diluted fourfold to lower urea concentration permitting trypsin digestion. Iodoacetamide (final concentration 5 mM) to alkylate cysteines and trypsin (220 ng/sample) was added to digest for another hour before the supernatant was transferred to another tube and digested overnight. The digest was stopped by addition of trifluoroacetic acid to 1% v/v and half of the peptide solution was further processed by desalting chromatography on three disks of C18 material using the STAGE‐tip format (Kulak *et al*, 2014). Briefly, STAGE‐tips were washed with 100 µl buffer B (50% v/v acetonitrile, 0.5% v/v acetic acid), conditioned with 100 µl methanol, washed twice with 100 µl buffer A (2% v/v acetonitrile, 0.5% v/v acetic acid), loaded with sample peptides, washed twice with 100 µl buffer A, and subjected to peptide elution by 60 µl of buffer B. The eluate was evaporated to dryness in a vacuum concentrator. Peptides were resuspended in 10 µl 2% v/v acetonitrile, 0.5% v/v acetic acid, 0.1% v/v trifluoroacetic acid and stored at −20°C. Peptide concentration was measured spectrophotometrically at 280 nm and 500 ng of peptide/sample were subjected to LC‐MS/MS analysis.

### MS data acquisition

Peptides were separated on an EASY‐nanoLC 1200 HPLC system (Thermo Fisher Scientific) via in‐house packed columns (75‐μm inner diameter, 50‐cm length, and 1.9‐μm C18 particles [Dr. Maisch GmbH]) in a gradient of buffer A (0.5% formic acid) to buffer B (80% acetonitrile, 0.5% formic acid). The gradient started at 5% B, increasing to 30% B in 40 min, further to 60% B in 4 min, to 95% B in 4 min, staying at 95% B for 4 min, decreasing to 5% B in 4 min and staying at 5% B for 4 min at a flow rate of 300 nl/min and a temperature of 60°C. A Quadrupole Orbitrap mass spectrometer (Q Exactive HF‐X for first dataset [wt TDP‐25 and TDP‐43], Exploris 480 for second dataset [wt and mut TDP‐25]; both Thermo Fisher Scientific) was directly coupled to the LC via a nano‐electrospray source. The mass spectrometers were operated in a data‐dependent mode. The survey scan range was set from 300 to 1,650 m/z, with a resolution of 60,000 at m/z 200. The most abundant isotope patterns (up to 12 or 10 on Q Exactive HF‐X and Exploris 480, respectively) with a charge of two to five were isolated and subjected to collision‐induced dissociation fragmentation (normalized collision energy of 27 or 30 on Q Exactive HF‐X and Exploris 480, respectively), an isolation window of 1.4 Th, and a MS/MS resolution of 15,000 at m/z 200. Dynamic exclusion to minimize re‐sequencing was set to 30 s.

### MS raw data processing

To process MS raw files, we employed the MaxQuant software (version 1.6.0.15 and 1.6.17.0 for the first and second dataset, respectively) (Cox & Mann, 2008), searching against the UniProtKB rat FASTA database using canonical and isoform protein sequences downloaded in March 2018. Default search parameters were utilized unless stated differently. In brief, tryptic peptides with a minimum length of 7 amino acids, a maximum mass of 4,600 Da, and two miscleavages at maximum were searched. Carbamidomethlyation was set as a fixed modification and methionine oxidation and protein N‐terminal acetylation as variable modifications. A maximum of five modifications per peptide was permitted. A false discovery rate (FDR) cutoff of 1% was applied at the peptide and protein level. The search feature “Match between runs,” which allows the transfer of peptide identifications in the absence of MS/MS‐based identification after nonlinear retention time alignment, was enabled with a maximum retention time window of 0.7 min. Protein abundances were normalized with the MaxLFQ label‐free normalization algorithm built into MaxQuant (Cox *et al*, 2014).

### Bioinformatic data analysis

The datasets were further processed and separately analyzed in the Perseus environment version 1.6.1.3 (Tyanova *et al*, 2016). Reverse (decoy) hits, proteins only identified by site, potential contaminants, and proteins not quantified in at least 65% of samples of at least one condition (construct and DIV) were removed. Protein abundances were log2‐transformed. Missing values for protein abundances were imputed from a normal distribution around the detection limit with a standard deviation of 0.3 times that of the quantified protein abundance distribution combining all samples and a downshift of 1.8 standard deviations. Volcano plot data were generated with the built‐in Perseus tool using a SAM statistic with an s0‐paramater of 0.1 to integrate the effect size and a permutation‐based FDR control set to 5% (Tusher *et al*, 2001). Proteins significantly (q‐value < 5%) associated with TDP‐25 or TDP‐43 were termed interactors and included in the further analysis.

To analyze the content of low‐complexity regions of neuronal TDP‐25/TDP‐43 interactors, we automatically queried the D2P2 database based on MaxQuant reported UniProt identifiers, using the first entry if multiple identifiers were reported in protein groups. Low complexity regions were defined as regions with a consensus of at least 6 of 9 analysis pipelines within D2P2 (Oates *et al*, 2013). Gene ontology (GO) analysis was performed focusing on the “biological process” categories of differentially immunoprecipitated proteins using clusterProfiler version 3.12 (Yu *et al*, 2012). A manual selection is shown to best represent the key pathways. The full list of significant GO terms is shown in Dataset EV2. The MS proteomics data have been deposited to the ProteomeXchange Consortium via the PRIDE partner repository with the dataset identifiers PXD024358 and PXD031083.

### Flow cytometry

Flow cytometry experiments were performed as described before (Guo *et al*, 2018). UbG76V‐GFP proteasome reporter cells were dissociated 72 h after transfection and analyzed with a Thermo Fisher Attune NxT Flow Cytometer (Thermo Fisher Scientific). To compensate for cross talk between GFP and tagRFP, HEK293 were transfected with tagRFP constructs only. Raw flow cytometry data were analyzed using FlowJo software (version 9.9, Treestar). The tagRFP channel was subdivided into different gates, corresponding to the log of fluorescence intensity of the transfected protein.

### Data visualization and statistical analysis

Data were visualized and statistically analyzed using R (version 3.6.1). Assumptions for parametric tests were analyzed with Shapiro‐Wilk test and Levene’s test. One‐way ANOVA with *post‐hoc* Tukey's HSD test was used as parametric test to compare more than two groups. Kruskal‐Wallis test and subsequent Pairwise Wilcoxon Rank Sum Tests with Benjamini‐Hochberg correction were used as non‐parametric tests. Data are plotted as mean ± standard deviation (SD) or box plots/violin plots. In the legends mean ± 95% CI is noted. *P*‐values within graphs are reported as follows: * denotes *P* ≤ 0.05, **P ≤ 0.01, ***P ≤ 0.001, *P* ≥ 0.05, ns.

## Author contribution

**Henrick Riemenschneider:** Conceptualization; Formal analysis; Investigation; Visualization; Methodology; Writing – original draft; Writing – review & editing. **Qiang Guo:** Conceptualization; Data curation; Formal analysis; Investigation; Visualization; Methodology; Writing – original draft; Writing – review & editing. **Jakob Bader:** Conceptualization; Data curation; Formal analysis; Investigation; Methodology. **Frédéric Frottin:** Formal analysis; Methodology. **Daniel Farny:** Methodology. **Gernot Kleinberger:** Methodology. **Christian Haass:** Supervision; Writing – review & editing. **Matthias Mann:** Funding acquisition. **F Ulrich Hartl:** Supervision; Funding acquisition; Writing – review & editing. **Wolfgang Baumeister:** Funding acquisition. **Mark S Hipp:** Conceptualization; Formal analysis; Supervision; Funding acquisition; Investigation; Writing – review & editing. **Felix Meissner:** Supervision. **Rubén Fernández‐Busnadiego:** Conceptualization; Formal analysis; Supervision; Funding acquisition; Investigation; Writing – original draft; Writing – review & editing. **Dieter Edbauer:** Conceptualization; Formal analysis; Supervision; Funding acquisition; Investigation; Visualization; Writing – original draft; Writing – review & editing.

In addition to the CRediT author contributions listed above, the contributions in detail are:

Conceptualisation, HR, QG, JB, MSH, RFB, and DE; Formal analysis, HR, QG, JB, FF, MSH, RFB, and DE; Methodology, HR, QG, JB, FF, DF, and GK; Investigation, HR, QG, JB, MSH, RFB, and DE; Data curation, HR, QG, and JB; Visualisation, HR, QG, and DE; Writing—original draft, HR, QG, RFB, and DE; Review & editing, HR, QG, CH, FUH, MSH, RFB, and DE; Supervision, CH, FUH, MSH, FM, RFB, and DE; Funding acquisition, MM, FUH, WB, MSH, RFB, and DE.

## Disclosure and competing interests statement

The authors declare that they have no conflict of interest.

## Supporting information



Expanded View Figures PDF

Dataset EV1

Dataset EV2

Dataset EV3

Movie EV1

## Data Availability

The datasets produced in this study are available in the following databases: Protein interaction AP‐MS data: PRIDE PXD024358 (http://www.ebi.ac.uk/pride/archive/projects/PXD024358), PRIDE PXD031083 (http://www.ebi.ac.uk/pride/archive/projects/PXD031083). cryoET Imaging datasets: EMDB EMD‐32216 (http://www.ebi.ac.uk/pdbe/entry/EMD‐32216; Subtomogram averaging of 26S proteasome from the cell with TDP‐25 inclusion) and EMDB EMD‐32217 (http://www.ebi.ac.uk/pdbe/entry/EMD‐32217; Electron tomogram of a rat primary neuron harboring TDP‐25 inclusion).
