# Author Correction: Gel-like inclusions of C-terminal fragments of TDP-43 sequester stalled proteasomes in neurons

**DOI:** 10.1038/s44319-024-00163-0

**Published:** 2024-06-07

**Authors:** Henrick Riemenschneider, Qiang Guo, Jakob Bader, Frédéric Frottin, Daniel Farny, Gernot Kleinberger, Christian Haass, Matthias Mann, F Ulrich Hartl, Wolfgang Baumeister, Mark S Hipp, Felix Meissner, Rubén Fernández-Busnadiego, Dieter Edbauer

**Affiliations:** 1 German Center for Neurodegenerative Diseases (DZNE), Munich, Feodor-Lynen-Str. 17, 81377 Munich, Germany; 2 Department of Molecular Structural Biology, Max Planck Institute of Biochemistry, 82152 Martinsried, Germany; 3 State Key Laboratory of Protein and Plant Gene Research, School of Life Sciences and Peking-Tsinghua Center for Life Sciences, Peking University, Beijing, 100871 China; 4 Department of Proteomics and Signal Transduction, Max Planck Institute for Biochemistry, Am Klopferspitz 18, 82152 Martinsried, Germany; 5 Department of Cellular Biochemistry, Max Planck Institute for Biochemistry, Am Klopferspitz 18, 82152 Martinsried, Germany; 6 Institute for Integrative Biology of the Cell (I2BC), Université Paris-Saclay, CEA, CNRS, 91198 Gif-sur-Yvette, France; 7 Chair of Metabolic Biochemistry, Biomedical Center (BMC), Faculty of Medicine, Ludwig-Maximilians-Universität, Munich, Germany; 8 Munich Cluster of Systems Neurology (SyNergy), Feodor-Lynen-Str. 17, 81377 Munich, Germany; 9 Department of Biomedical Sciences of Cells and Systems, University Medical Center Groningen, University of Groningen, Antonius Deusinglaan 1, 9713 AV Groningen, The Netherlands; 10 School of Medicine and Health Sciences, Carl von Ossietzky University Oldenburg, Oldenburg, Germany; 11 Institute of Innate Immunity, Department of Systems Immunology and Proteomics, Medical Faculty, University of Bonn, Bonn, Germany; 12 Institute of Neuropathology, University Medical Center Göttingen, 37099 Göttingen, Germany; 13 Cluster of Excellence “Multiscale Bioimaging: from Molecular Machines to Networks of Excitable Cells” (MBExC), University of Göttingen, 37075 Göttingen, Germany; 14 Graduate School of Systemic Neurosciences (GSN), Ludwig-Maximilians-University Munich, 81377 Munich, Germany

## Abstract

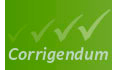

**Correction to:** *EMBO Reports* (2022) 23:e53890. 10.15252/embr.202153890 | Published online 19 April 2022

The Acknowledgements section is corrected.

D.E., C.H., M.S.H., F.U.H. and R.F.-B. acknowledge funding from the Deutsche Forschungsgemeinschaft (DFG, German Research Foundation) through Germany’s Excellence Strategy - EXC 2067/1- 390729940 (R.F.-B.) and EXC 2145 – 390857198 (C.H., F.U.H., M.S.H. and D.E.)

Is corrected to: Changes in bold.

D.E., C.H., M.S.H., F.U.H., and R.F.-B. acknowledge funding from the Deutsche Forschungsgemeinschaft (DFG, German Research Foundation) **through SFB1286/A12 (R. F.-B.) as well as Germany’s Excellence Strategy - EXC 2067/1- 390729940 (R.F.-B.) and EXC 2145 – 390857198 (C.H., F.U.H., M.S.H., and D.E.)**

