# Supplementary material for: Gel‐like inclusions of C‐terminal fragments of TDP‐43 sequester stalled proteasomes in neurons
Source: EMBO Rep. 2022 Apr 19;23(6):e53890. doi: 10.15252/embr.202153890 (PMC9171420; doi:10.15252/embr.202153890)
Supplement: Supplementary file 1 — Expanded View Figures PDF [file EMBR-23-e53890-s005.pdf]

Expanded View Figures

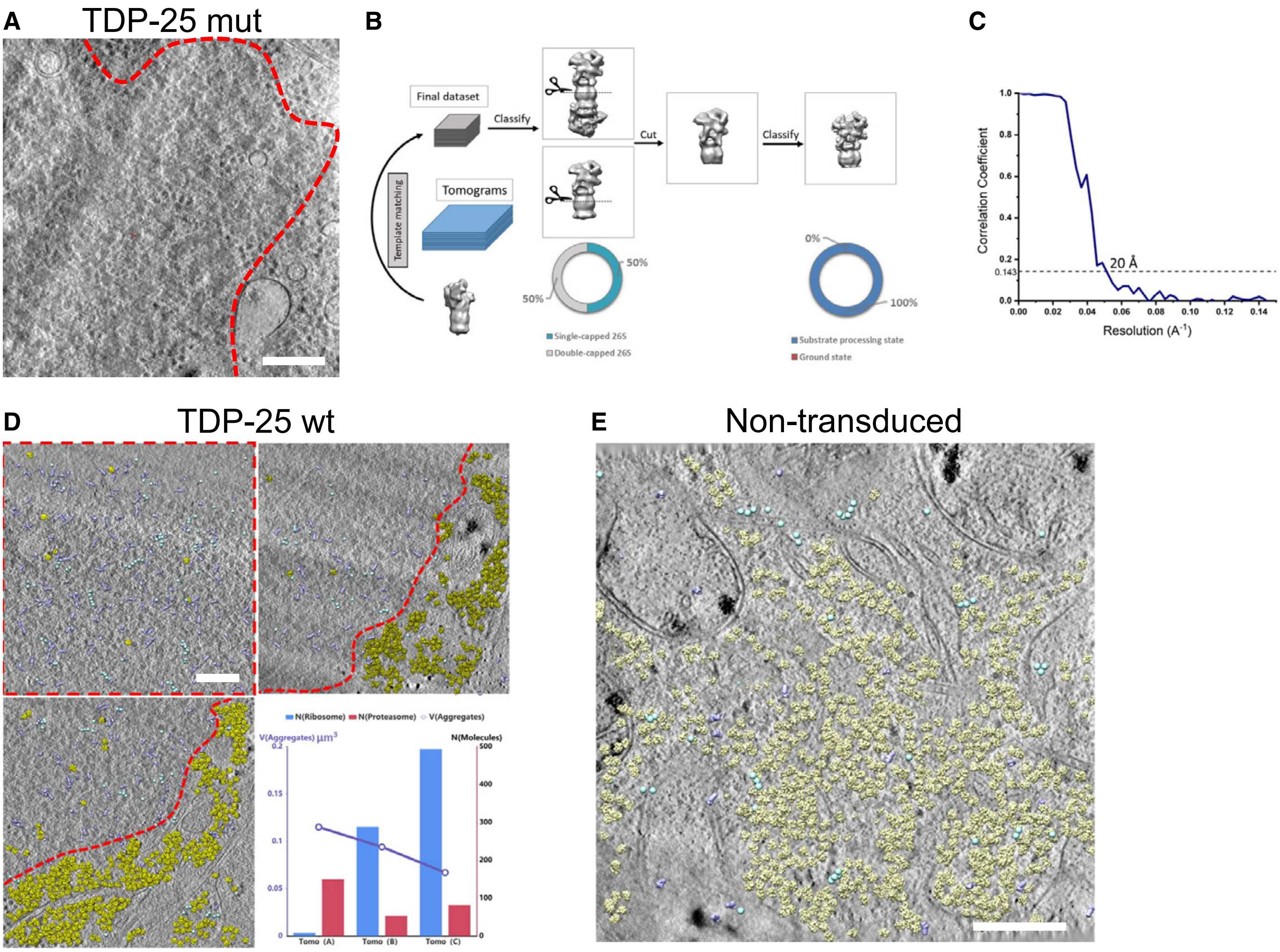

Figure EV1. Proteasomes are enriched in TDP-25 inclusions.

A Tomographic slice of an aggregate within a neuron transduced with GFP-TDP-25 mutant construct (DIV5+8). Red dotted line segments aggregate area. Scale bar = 200 nm.

B Workflow of subtomogram averaging and classification. Subtomograms were identified using a low-resolution single-capped proteasome as template. All proteasomes were firstly classified into single-capped or double-capped. To further analyze the conformation of the regulatory particles, all proteasomes were cut *in silico* between the β-rings of the core particle, resulting in two independent particles for double-capped ones. 50% of the particles were assigned to the single and double-capped classes, respectively. Cut out regulatory particles were merged and subjected to a further round of classification. All regulatory particles were assigned to substrate processing conformations. However, due to the uncertainties inherent to the classification procedure, it is possible that a small fraction of particles adopted other conformations.

C Gold-standard Fourier shell correlation curve of the proteasome structure showing a resolution of 20 Å.

D Molecular mapping in three tomograms of GFP-TDP-25 wild-type inclusions in transduced neurons (DIV5+8). Regions containing GFP-TDP-25 are outlined in red. For the whole tomogram, proteasomes (purple), TRiC (cyan) and ribosomes (yellow) are mapped to their original positions and orientations using the information from template matching and subtomogram averaging. The numbers of proteasomes and ribosomes detected in the tomograms are plotted versus the volume of the tomogram. Scale bar = 200 nm.

E Molecular mapping in a tomogram recorded on a non-transduced control neuron, with proteasomes (purple), ribosomes (yellow) and TRiC (cyan) plotted as in Fig EV1D. This tomogram contains 24 proteasomes, 1,222 ribosomes and 73 TRiC molecules. Scale bar = 200 nm.

© 2022 The Authors

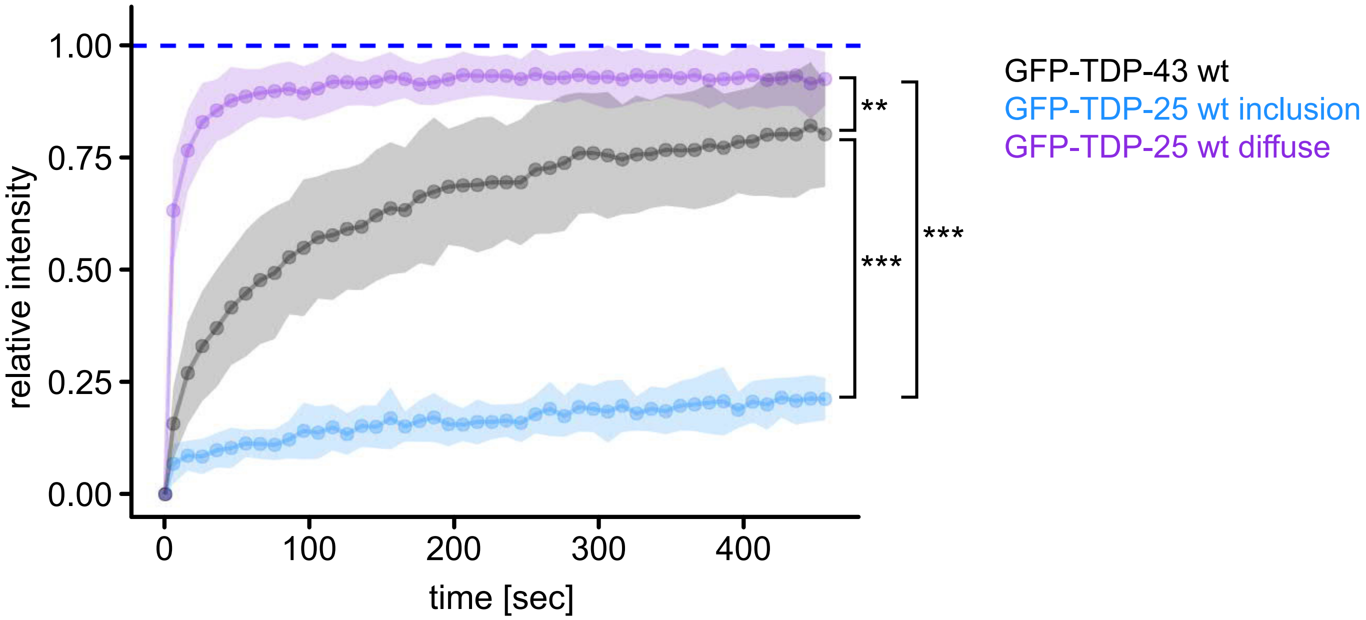

Figure EV2. TDP-25 inclusions are less mobile than TDP-43.

Rat primary hippocampal neurons were transduced with GFP-TDP-25 wild-type (DIV5+8) or GFP-TDP-43 wild-type (DIV9+4) and analyzed by FRAP. The resulting normalized FRAP curves (relative fluorescence intensity over time) with values representing means ± SD are shown. Comparison of the averaged recovery fractions of the last six timepoints obtained from three independent experiments: GFP-TDP-43 (0.802 ± 0.06, mean ± CI, n = 20 cells) vs. GFP-TDP-25 inclusion (0.209 ± 0.019, n = 18 cells) vs. GFP-TDP-25 diffuse (0.926 ± 0.045, n = 10 cells): H(1) = 36.295, df = 2, P = 1.314*10^{-8}, η^2[H] = 0.762, Kruskal-Wallis Test. GFP-TDP-43 vs. GFP-TDP-25 inclusion: ***P = 1.8*10^{-10}, GFP-TDP-43 vs. GFP-TDP-25 diffuse: **P = 0.0033, GFP-TDP-25 inclusion vs. GFP-TDP-25 diffuse: ***P = 2.3*10^{-7}, Pairwise Wilcoxon Rank Sum Tests with Benjamini-Hochberg correction.

 © 2022 The Authors

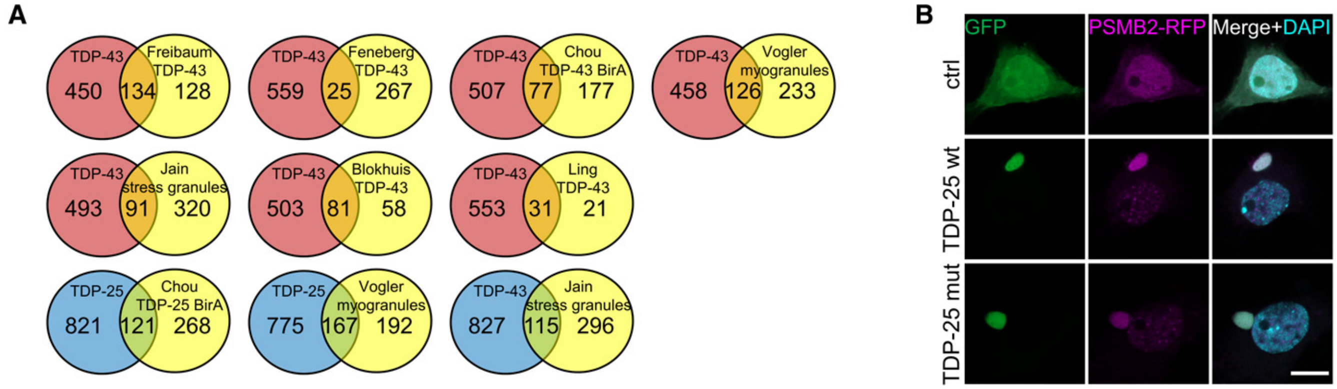

C

comparison (p_{adj}) / fluorescence intensity bin	5	6	7	8	9	10	11	12	13	14	15	16	17	18	19	20	21
TDP-25 mut - RFP	0.057	0.041	0.041	0.041	0.036	0.036	0.036	0.041	0.036	0.036	0.036	0.036	0.036	0.041	0.036	0.036	0.036
TDP-25 wt - RFP	0.057	0.041	0.041	0.041	0.036	0.036	0.036	0.041	0.036	0.036	0.036	0.036	0.036	0.041	0.036	0.036	0.036
TDP-43 mut - RFP	0.286	0.143	0.041	0.041	0.036	0.036	0.036	0.041	0.036	0.036	0.036	0.036	0.036	0.041	0.036	0.036	0.036
TDP-43 wt - RFP	0.381	0.222	0.127	0.686	0.036	0.036	0.036	0.200	0.036	0.036	0.036	0.036	0.036	0.071	0.036	0.036	0.036
TDP-25 mut - TDP-25 wt	0.057	0.041	0.071	0.143	0.127	0.114	0.114	0.200	0.200	0.127	0.114	0.127	0.114	0.127	0.200	0.127	0.200
TDP-25 mut - TDP-43 mut	0.095	0.041	0.041	0.041	0.036	0.036	0.036	0.041	0.036	0.036	0.036	0.036	0.036	0.041	0.036	0.036	0.036
TDP-25 wt - TDP-43 wt	0.381	0.041	0.041	0.041	0.036	0.036	0.036	0.041	0.036	0.036	0.036	0.036	0.036	0.041	0.036	0.036	0.036
TDP-43 mut - TDP-43 wt	0.686	0.343	0.200	0.222	0.200	0.114	0.114	0.200	0.200	0.200	0.114	0.343	0.063	0.200	0.127	0.200	0.063

comparison (p_{adj}) / fluorescence intensity bin	22	23	24	25	26	27	28	29	30	31	32	33	34	35	36	37	38
TDP-25 mut - RFP	0.032	0.036	0.036	0.036	0.036	0.036	0.036	0.036	0.041	0.036	0.041	0.048	0.048	0.041	0.041	0.036	0.036
TDP-25 wt - RFP	0.032	0.036	0.036	0.036	0.036	0.036	0.036	0.036	0.041	0.036	0.041	0.048	0.048	0.041	0.041	0.036	0.036
TDP-43 mut - RFP	0.032	0.036	0.036	0.036	0.036	0.036	0.036	0.036	0.041	0.036	0.041	0.048	0.048	0.041	0.041	0.036	0.036
TDP-43 wt - RFP	0.032	0.036	0.036	0.036	0.036	0.036	0.036	0.036	0.041	0.036	0.041	0.048	0.048	0.041	0.041	0.036	0.036
TDP-25 mut - TDP-25 wt	0.114	0.200	0.127	0.127	0.127	0.127	0.063	0.063	0.114	0.036	0.041	0.048	0.048	0.041	0.041	0.036	0.036
TDP-25 mut - TDP-43 mut	0.032	0.036	0.036	0.036	0.036	0.036	0.036	0.036	0.041	0.036	0.041	0.082	0.486	0.071	0.071	0.036	0.036
TDP-25 wt - TDP-43 wt	0.032	0.036	0.036	0.036	0.036	0.036	0.036	0.036	0.063	0.200	0.686	0.222	0.048	0.041	0.041	0.036	0.036
TDP-43 mut - TDP-43 wt	0.032	0.127	0.200	0.200	0.343	0.343	0.114	0.114	0.063	0.063	0.127	0.143	0.163	0.127	0.127	0.200	0.057

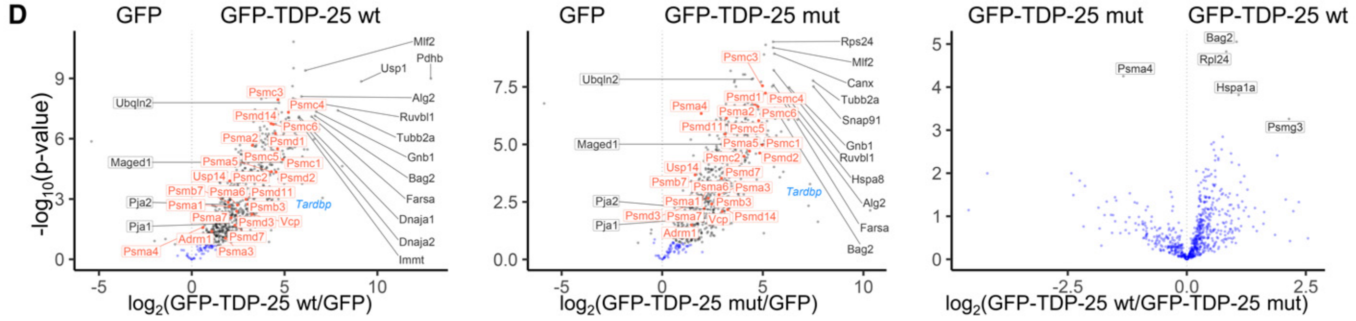

Figure EV3. Additional interactome analyses.

A Proteins identified in the GFP-TDP-43 interactome from Fig 3A (uniquely or in a cluster) were compared to published datasets (Freibaum *et al*, 2010; Ling *et al*, 2010; Blokhuis *et al*, 2016; Chou *et al*, 2018; Feneberg *et al*, 2020). LOC and RGD gene names that usually represent minor components of the identified clusters were removed, because such entries could not be mapped based on gene names between species. A core set of 32 proteins from our TDP-43 interactome was found in four (DDX3X, DHX9, ELAVL1, HNRNPA0, HNRNPD, HNRNPH1, HNRNPL, HNRNPM, HNRNPR, ILF2, MATR3, PABPC4, RALY, YBX1) or three (DDX5, DDX6, EFTUD2, EIF3A, HNRNPA3, HNRNPAB, HNRNPC, HNRNPK, HNRNPU, HNRNPUL2, ILF3, NONO, NOP56, NOP58, PABPC1, SNRNP200, SNRPA1, SSB) interactome datasets (Freibaum *et al*, 2010; Ling *et al*, 2010; Blokhuis *et al*, 2016; Feneberg *et al*, 2020). There was also substantial overlap of our TDP-25 interactome with a BirA proximity labeling dataset for TDP-25 (Chou *et al*, 2018), the stress granule proteome (Jain *et al*, 2016) and TDP-43 myogranules in regenerating muscle (Vogler *et al*, 2018).

B Colocalization of proteasomes with TDP-25 inclusions. Primary rat hippocampal neurons were co-transduced with lentivirus encoding for GFP-tagged TDP-25 variants or GFP and PSMB2-tagRFP lentivirus on day 5. Immunofluorescence images were taken 8 days after transduction (DIV5+8). Counterstain to label nuclei (DAPI). Scale bar = 10 μm.

C Statistical analysis for Fig 3F. Pairwise comparisons using Wilcoxon Rank Sum Tests with Benjamini-Hochberg correction were performed for each tagRFP intensity bin respectively. Comparisons with $P < 0.05$ are highlighted in green.

D Interactome analysis of TDP-25 mutant. Rat primary cortical neurons were transduced with GFP-TDP-25 wild-type ($n = 5$), GFP-TDP-25 mutant ($n = 5$) or GFP ($n = 4$) at DIV5 and harvested at DIV5+8. Immunoprecipitates were analyzed by LC-MS/MS. Volcano plots indicate enrichment and statistical significance (gray dots: FDR-corrected $P < 0.05$). Proteins associated with GO term “proteasome complex” (GO:0000502) are labeled in orange. Full data are listed in Dataset EV3. This data was obtained from a second experiment independent from Fig 2A.
