# Supplementary material for: Gel‐like inclusions of C‐terminal fragments of TDP‐43 sequester stalled proteasomes in neurons
Source: EMBO Rep. 2022 Apr 19;23(6):e53890. doi: 10.15252/embr.202153890 (PMC9171420; doi:10.15252/embr.202153890)
Supplement: Supplementary file 5 — Movie EV1 [file EMBR-23-e53890-s006.zip › MovieEV1/_MovieEV1_legend.docx]

**Movie EV1. Mapping Macromolecules to TDP-25 Inclusions, Related to Figure 2**

Tomographic volume and 3D rendering of the neuronal GFP-TDP-25 inclusion depicted in Fig 1C/D. GFP-TDP-25 (red), 26S proteasomes (violet), ribosomes (yellow), TRiC/CCT chaperonins (green), mitochondria

in white, ER in pink and other vesicles in light yellow. The macromolecules are mapped to their original

positions and orientations, computationally determined by template matching and subtomogram

averaging. Proteasomes are enriched within the GFP-TDP-25 inclusions, while ribosomes are mostly

found in its periphery.
