# Supplementary material for: Gel‐like inclusions of C‐terminal fragments of TDP‐43 sequester stalled proteasomes in neurons
Source: EMBO Rep. 2022 Apr 19;23(6):e53890. doi: 10.15252/embr.202153890 (PMC9171420; doi:10.15252/embr.202153890)
Supplement: Supplementary file 6 [file EMBR-23-e53890-s001.pdf]

EMBO reports

Gel-like inclusions of C-terminal fragments of TDP-43 sequester stalled proteasomes in neurons

Henrick Riemenschneider, Qiang Guo, Jakob Bader, Frédéric Frottin, Daniel Farny, Gernot Kleinberger, Christian Haass, Matthias Mann, F. Ulrich Hartl, Wolfgang Baumeister, Mark Hipp, Felix Meissner, Rubén Fernández-Busnadiego, and Dieter Edbauer
DOI: 10.15252/embr.202153890

Corresponding author(s): Dieter Edbauer (Dieter.Edbauer@dzne.de) , Rubén Fernández-Busnadiego (ruben.fernandezbusnadiego@med.uni-goettingen.de)

Review Timeline:

Submission Date:	26th Aug 21
Editorial Decision:	12th Oct 21
Revision Received:	21st Dec 21
Editorial Decision:	14th Mar 22
Revision Received:	25th Mar 22
Accepted:	30th Mar 22

Editor: Esther Schnapp

Transaction Report:

(Note: With the exception of the correction of typographical or spelling errors that could be a source of ambiguity, letters and reports are not edited. Depending on transfer agreements, referee reports obtained elsewhere may or may not be included in this compilation. Referee reports are anonymous unless the Referee chooses to sign their reports.)

Dear Dieter,

Thank you for the submission of your manuscript to EMBO reports. We have now received the full set of referee reports that is pasted below.

As you will see, the referees acknowledge that the findings are potentially interesting. However, they also raise several concerns, and I think all should be addressed. Especially the physiological relevance of the TDP-25 peptide should to be strengthened. If you disagree or have any other comments, we can also discuss the revisions in a video chat, if you like.

I would thus like to invite you to revise your manuscript with the understanding that the referee concerns must be fully addressed and their suggestions taken on board. Please address all referee concerns in a complete point-by-point response. Acceptance of the manuscript will depend on a positive outcome of a second round of review. It is EMBO reports policy to allow a single round of major revision only and acceptance or rejection of the manuscript will therefore depend on the completeness of your responses included in the next, final version of the manuscript.

Revised manuscripts should be submitted within three months of a request for revision; they will otherwise be treated as new submissions. Please contact us if a 3-months time frame is not sufficient for the revisions so that we can discuss this further. You can either publish the study as a short report or as a full article. For short reports, the revised manuscript should not exceed 27,000 characters (including spaces but excluding materials & methods and references) and 5 main plus 5 expanded view figures. The results and discussion sections must further be combined, which will help to shorten the manuscript text by eliminating some redundancy that is inevitable when discussing the same experiments twice. For a normal article there are no length limitations, but it should have more than 5 main figures and the results and discussion sections must be separate. In both cases, the entire materials and methods must be included in the main manuscript file.

IMPORTANT NOTE: we perform an initial quality control of all revised manuscripts before re-review. Your manuscript will FAIL this control and the handling will be DELAYED if the following APPLIES:
1) A data availability section providing access to data deposited in public databases is missing. If you have not deposited any data, please add a sentence to the data availability section that explains that.
2) Your manuscript contains statistics and error bars based on n=2. Please use scatter blots in these cases. No statistics should be calculated if n=2.

When submitting your revised manuscript, please carefully review the instructions that follow below. Failure to include requested items will delay the evaluation of your revision.

1) a .docx formatted version of the manuscript text (including legends for main figures, EV figures and tables). Please make sure that the changes are highlighted to be clearly visible.

2) individual production quality figure files as .eps, .tif, .jpg (one file per figure). See https://wol-prod-cdn.literatumonline.com/pb-assets/embo-site/EMBOPress_Figure_Guidelines_061115-1561436025777.pdf for more info on how to prepare your figures.

3) We replaced Supplementary Information with Expanded View (EV) Figures and Tables that are collapsible/expandable online. A maximum of 5 EV Figures can be typeset. EV Figures should be cited as 'Figure EV1, Figure EV2" etc... in the text and their respective legends should be included in the main text after the legends of regular figures.

- For the figures that you do NOT wish to display as Expanded View figures, they should be bundled together with their legends in a single PDF file called *Appendix*, which should start with a short Table of Content. Appendix figures should be referred to in the main text as: "Appendix Figure S1, Appendix Figure S2" etc. See detailed instructions regarding expanded view here: <https://www.embopress.org/page/journal/14693178/authorguide#expandedview>

- Additional Tables/Datasets should be labeled and referred to as Table EV1, Dataset EV1, etc. Legends have to be provided in a separate tab in case of .xls files. Alternatively, the legend can be supplied as a separate text file (README) and zipped together with the Table/Dataset file.

4) a .docx formatted letter INCLUDING the reviewers' reports and your detailed point-by-point responses to their comments. As part of the EMBO Press transparent editorial process, the point-by-point response is part of the Review Process File (RPF), which will be published alongside your paper.

5) a complete author checklist, which you can download from our author guidelines <https://www.embopress.org/page/journal/14693178/authorguide>. Please insert information in the checklist that is also reflected in the manuscript. The completed author checklist will also be part of the RPF.

6) Please note that all corresponding authors are required to supply an ORCID ID for their name upon submission of a revised

manuscript (<https://orcid.org/>). Please find instructions on how to link your ORCID ID to your account in our manuscript tracking system in our Author guidelines
<https://www.embopress.org/page/journal/14693178/authorguide#authorshipguidelines>

7) Before submitting your revision, primary datasets produced in this study need to be deposited in an appropriate public database (see https://www.embopress.org/page/journal/14693178/authorguide#datadeposition). Please remember to provide a reviewer password if the datasets are not yet public. The accession numbers and database should be listed in a formal "Data Availability" section placed after Materials & Method (see also https://www.embopress.org/page/journal/14693178/authorguide#datadeposition). Please note that the Data Availability Section is restricted to new primary data that are part of this study. * Note - All links should resolve to a page where the data can be accessed. *
If your study has not produced novel datasets, please mention this fact in the Data Availability Section.

8) We would also encourage you to include the source data for figure panels that show essential data. Numerical data should be provided as individual .xls or .csv files (including a tab describing the data). For blots or microscopy, uncropped images should be submitted (using a zip archive if multiple images need to be supplied for one panel). Additional information on source data and instruction on how to label the files are available at
<https://www.embopress.org/page/journal/14693178/authorguide#sourcedata>.

9) Our journal also encourages inclusion of *data citations in the reference list* to directly cite datasets that were re-used and obtained from public databases. Data citations in the article text are distinct from normal bibliographical citations and should directly link to the database records from which the data can be accessed. In the main text, data citations are formatted as follows: "Data ref: Smith et al, 2001" or "Data ref: NCBI Sequence Read Archive PRJNA342805, 2017". In the Reference list, data citations must be labeled with "[DATASET]". A data reference must provide the database name, accession number/identifiers and a resolvable link to the landing page from which the data can be accessed at the end of the reference. Further instructions are available at https://www.embopress.org/page/journal/14693178/authorguide#referencesformat

10) Regarding data quantification (see Figure Legends:
https://www.embopress.org/page/journal/14693178/authorguide#figureformat)

The following points must be specified in each figure legend:

- the name of the statistical test used to generate error bars and P values,

- the number (n) of independent experiments (please specify technical or biological replicates) underlying each data point,

- the nature of the bars and error bars (s.d., s.e.m.),

- If the data are obtained from n {less than or equal to} 2, use scatter blots showing the individual data points.

Discussion of statistical methodology can be reported in the materials and methods section, but figure legends should contain a basic description of n, P and the test applied.

- Please also include scale bars in all microscopy images.

We would also welcome the submission of cover suggestions, or motifs to be used by our Graphics Illustrator in designing a cover.

As part of the EMBO publication's Transparent Editorial Process, EMBO reports publishes online a Review Process File (RPF) to accompany accepted manuscripts. This File will be published in conjunction with your paper and will include the referee reports, your point-by-point response and all pertinent correspondence relating to the manuscript.

You are able to opt out of this by letting the editorial office know (emboreports@embo.org). If you do opt out, the Review Process File link will point to the following statement: "No Review Process File is available with this article, as the authors have chosen not to make the review process public in this case."

I look forward to seeing a revised form of your manuscript when it is ready. Please use this link to submit your revision:
https://embor.msubmit.net/cgi-bin/main.plex

Best wishes,
Esther

Esther Schnapp, PhD
Senior Editor

Referee #1:

In this work, Riemenschneider have investigated the type of inclusions formed by a toxic C-terminal fragment of TDP-43 called TDP-25. Until this moment, the toxic mechanisms promoted by this fragment have never been clarified. In this work, the authors have combined several state-of-the-art techniques to provide an explanation. From their studies, the authors have concluded that TDP-25 toxicity could principally stems from an impairment of the cellular proteasomal machinery. In general, the experiments have been performed to very high technical standards and results are convincing. A few additions/clarifications would nonetheless help to strengthen the conclusions of the manuscript:

- The data in Figure 3F suggest that at high expression levels, TDP-25 mut impaired overall protein degradation significantly more than TDP-25 wt. Looking at the curves in this figure, this certainly seems to be the case. However, no significance values are reported in the inset with regards to tagRFP-TDP-25 wt vs. tagRFP-TDP-25 mut, they are only referred to tagRFP. Have these values been calculated?. In this experiment, it would have been interesting to see how these fragments compare with the TDP-43 wt protein.
- Most interestingly, the considerable difference between TDP-25wt and TDP-25mut in Figure 2D, compared to the high similarity between these two fragments in the structural and photobleaching experiments, would suggest that the explanation may reside in the protein interaction profile. Have the authors considered performing a proteomic experiment with GFP-TDP-25 mut and compared the profile with the one obtained using GFP-TDP-25 wt?
- Related to this, one of the most striking data from this study is that compared to TDP-43 wt the TDP-25 fragment loses only 72 interactions but gains more than 400 ones. However, the TDP-25 sequence is completely present in the TDP-43 wt sequence and this raises the question how this can occur. This aspect is not discussed in the manuscript and, although additional experiments may be beyond the scope of this work, some speculation could be added to improve Conclusions. For example, could the structures in Fig.1E be particularly "sticky" for proteasome components?.
- Finally, one important aspect that has not been addressed in this study is the potential physiological importance of this TDP-25 mediated proteasomal inhibition in physiological conditions. It must be noted, in fact, that the presence of the 25kDa fragment in patient neuronal tissue does not seem to occur at the very high levels used in this study. Indeed, in all western blots from ALS/FTLD-TDP patients the presence of the 25kDa (and sometimes 35kDa) fragments does not seem to appreciably reduce the amount of full length TDP-43 present in the sample. This raises the question whether the proteosomal inhibition mediated by TDP-25 could represent a pathological effect even when present in lesser quantities. At the very least, this is a caveat that should be discussed in Conclusions. Ideally, the authors could see whether the structures identified in the CryoEM experiments or increased proteasomal inhibition also exist in cells where 25 kDa production can be induced to somewhat similar pathological levels using various chemicals (ie. Dexamethasone, L-BMAA, para-Chloroamphetamine).

Referee #2:

This is a well-written paper to use three pronged approaches of cryo-ET, mass spec and functional assays to tackle a complex neurodegeneration problem on where and how the TDP-25 impact on cellular functions. The conclusion that proteasome dysfunction is a hallmark of ALS/FTD pathogenesis is not surprising and opens more avenues in therapeutic strategies. However, I have a few technical questions on the cryoET analysis that needs to be clarified in the text or the supplement.

1. How do they pick the aggregates (i.e., the annotated red features) in Figure 1D to be GFP-TDP-25? They are variable sizes and shapes. Was a control done from cells without GFP-TDP-25?

2. Are the TDP-25 inclusions localized only in certain region of the cells?

3. Is there any cryo-ET structural difference between the wildtype and mutant GFP-TDP-25 inclusions?

4. I am concerned about using template to pick ribosomes, proteosomes and TRiC. Have they carried out independent approach to validate the picked proteosomes to be in the same structural state? Again, a side by side control experiment without TDP-25 present cell would be great.

5. What are the particle statistics to derive the plot in Supp Figure 1C?

6. What are the cryoET data statistics before the conclusions are made. How do the plots shown in Supplement Figure 1D compared between cells without and with TDP-25 transduction.

7. It is necessary that at least one representative tomogram reconstruction is deposited to the EMDB for the public use. In addition, the subtomogram average of the proteasome along with the FSC plot should also be deposited.

Referee #3:

The manuscript by Riemenschneider et al. describes the characterisation of aggregation of a truncated version of TDP-25 in to inclusions. The paper reports the formation of solidified structures that appear to be non-fibrillar and associate with stalled conformation of proteosomes.
The paper is well written but the shortened format has lead to some questions regarding the approaches that are a unclear to the reader. The paper includes a large amount of electron tomography but the description of it really enough lacks detail. There is a lack of information on repeats, statistics and analysis methods.

Figure 1A shows a single example of co-localisation of TDP25 (wt or mutant) with P-TDP43. There is no quantification or statistics here. Figure 1B shows a single WB again with no quantification or stats.
In Figure 1C, how have TDP25 containing inclusions been identified in the cells? It would be helpful to include further explanation of the annotation process for ET. These additional details are necessary to give confidence for the reported results.

Minor :
1. the abstract states that TDP43 correlates with FTD but in the introduction, it is more correctly stated that it correlates with 45% of FTD, so the abstract should be amended (some cases of FTD).

2. This sentence in the introduction needs attention:
"Here, we aimed to elucidate gain-of-function mechanisms as well as the structure of cytoplasmic TDP-43 aggregates found in sporadic and most genetic ALS/FTD cases we focused on the aggregation-prone TDP-25 fragment (residues 220-414 of full length human TDP-43) (Neumann et al, 2006; Zhang et al, 2009) using a pipeline of cryo-ET, proteomics and functional assays"

Overall, this is an interesting paper but I feel unsure of the use and significance of the truncation version of TDP43. The authors may need to defend their use of this fragment further for the physiological relevance of their findings for ALS and FTD that involves TDP43

Referee #1:

In this work, Riemenschneider have investigated the type of inclusions formed by a toxic C-terminal fragment of TDP-43 called TDP-25. Until this moment, the toxic mechanisms promoted by this fragment have never been clarified. In this work, the authors have combined several state-of-the-art techniques to provide an explanation. From their studies, the authors have concluded that TDP-25 toxicity could principally stems from an impairment of the cellular proteasomal machinery. In general, the experiments have been performed to very high technical standards and results are convincing. A few additions/clarifications would nonetheless help to strengthen the conclusions of the manuscript:

- The data in Figure 3F suggest that at high expression levels, TDP-25 mut impaired overall protein degradation significantly more than TDP-25 wt. Looking at the curves in this figure, this certainly seems to be the case. However, no significance values are reported in the inset with regards to tagRFP-TDP-25 wt vs. tagRFP-TDP-25 mut, they are only referred to tagRFP. Have these values been calculated? In this experiment, it would have been interesting to see how these fragments compare with the TDP-43 wt protein.

We thank the reviewer for this comment. We added the wild-type and mutant full-length protein (tagRFP-TDP-43) in the revised Figure 3F and provide a detailed statistical analysis in the new Fig. EV3C to maintain clarity with the higher number of comparisons. While low levels of RFP-TDP-43 have minimal effect compared to the RFP control, high level expression inhibit proteasomal processing of the reporter construct, which is consistent with proteasomal enrichment also in the TDP-43 full length interactome (see Figure 3A), albeit to a lesser extent than in the TDP-25 interactome. Interestingly, a full length TDP-43 containing the eight ALS-causing mutations further increased the reporter levels at high expression levels, without reaching statistical significance.

- Most interestingly, the considerable difference between TDP-25wt and TDP-25mut in Figure 2D, compared to the high similarity between these two fragments in the structural and photobleaching experiments, would suggest that the explanation may reside in the protein interaction profile. Have the authors considered performing a proteomic experiment with GFP-TDP-25 mut and compared the profile with the one obtained using GFP-TDP-25 wt?

We assume this comment refers to Figure 3F. We followed this excellent suggestion and compared the interactomes of wild-type and mutant TDP-25 (extended Figure EV3D and Table S3). In these replicate experiments the interactome of wild-type TDP-25 was strongly enriched in proteasomal subunits, which convincingly reproduces our initial findings. Furthermore, the interaction profile of TDP-25 mutant also revealed a prominent enrichment of proteasomal subunits.

While the overall interaction profiles were comparable, mutant TDP-25 interacted weaker with Hspa1a, a member of the Hsp70 family, and Bag2, a Hsp70/Hsc70 interacting co-chaperone, but stronger with the proteasome core subunit Psma4 (new Figure EV3D). Thus, the effects of the ALS mutations may be indirect, e.g. through proteins co-partitioning into the inclusions without direct interaction with TDP-25. Moreover, introducing the same mutations into full length TDP-43 also impaired proteostasis more than wild-type TDP-43 (revised Fig 3F).

- Related to this, one of the most striking data from this study is that compared to TDP-43 wt the TDP-25 fragment loses only 72 interactions but gains more than 400 ones. However, the TDP-25 sequence is completely present in the TDP-43 wt sequence and this raises the question how this can occur. This aspect is not discussed in the manuscript and, although additional experiments may be

beyond the scope of this work, some speculation could be added to improve Conclusions. For example, could the structures in Fig.1E be particularly "sticky" for proteasome components?

It is indeed counterintuitive that a protein fragment has more interactors than the full-length protein. However, this effect could be explained by co-partitioning of other proteins into the gel-like inclusions. Moreover, protein misfolding rapidly recruits the UPS machinery and other proteins that are also ubiquitinated (e.g. Gottlieb et al, JBC 2019). Finally, our data is consistent with findings from Chou et al (Nat Neuro 2017) using proximity labeling.

The extra density in Fig 1E likely reflects substrates or adaptor proteins as mentioned in the result section. We do not have structures of proteasomes in TDP-43 overexpressing cells and thus cannot tell whether this is TDP-25 specific material. However, we noticed similar extra densities in poly-GA expressing cells (Guo et al, Cell 2018). Although the resolution of the extra density does not allow its molecular identification, it is localized to a specific region of the proteasome, suggesting that the interaction is specific at least to some extent.

- Finally, one important aspect that has not been addressed in this study is the potential physiological importance of this TDP-25 mediated proteasomal inhibition in physiological conditions. It must be noted, in fact, that the presence of the 25kDa fragment in patient neuronal tissue does not seem to occur at the very high levels used in this study. Indeed, in all western blots from ALS/FTLD-TDP patients the presence of the 25kDa (and sometimes 35kDa) fragments does not seem to appreciably reduce the amount of full length TDP-43 present in the sample. This raises the question whether the proteosomal inhibition mediated by TDP-25 could represent a pathological effect even when present in lesser quantities. At the very least, this is a caveat that should be discussed in Conclusions. Ideally, the authors could see whether the structures identified in the CryoEM experiments or increased proteasomal inhibition also exist in cells where 25 kDa production can be induced to somewhat similar pathological levels using various chemicals (ie. Dexamethasone, L-BMAA, para-Chloroamphetamine).

We thank the reviewer for this comment and acknowledge the value of the suggested experiment. The research community still lacks a sophisticated model system that shows C-terminal fragmentation without relying on overexpression of TDP-43 species. Despite our efforts, we couldn't detect any C-terminal TDP-43 fragments upon treatment with various concentrations of Dexamethasone (= Dexam., toxic at 500µM), L-BMAA, BAPTA-AM (toxic at 100µM), EDTA (toxic at 2.5-100mM) or 15d-PGJ2 (toxic at 20µM) for up to 24h in the UbG76V-GFP reporter HEK293 cell line (see Figure below). The ordered para-Chloroamphetamine was never delivered and could not be tested. Therefore, we unfortunately could not assess the effect of "endogenous" TDP-25 levels on proteasome activity. Moreover, we didn't test compounds in primary neurons, because they would presumably induce very small aggregates (similar to MG132 treatment, Khosravi et al, EMBO J 2020), which are beyond the CLEM resolution and would not allow analysis by cryo-ET.

However, comparison with RFP and the newly added RFP-TDP-43 clearly shows that RFP-TDP-25 increases reporter levels in all but the two lowest intensity bins, which strongly argues for proteasome inhibition also at low expression levels, although the difference between wild-type and mutant TDP-25 only becomes apparent at high expression levels (revised Fig 3F and new EV3C). We emphasized this point in the revised manuscript.

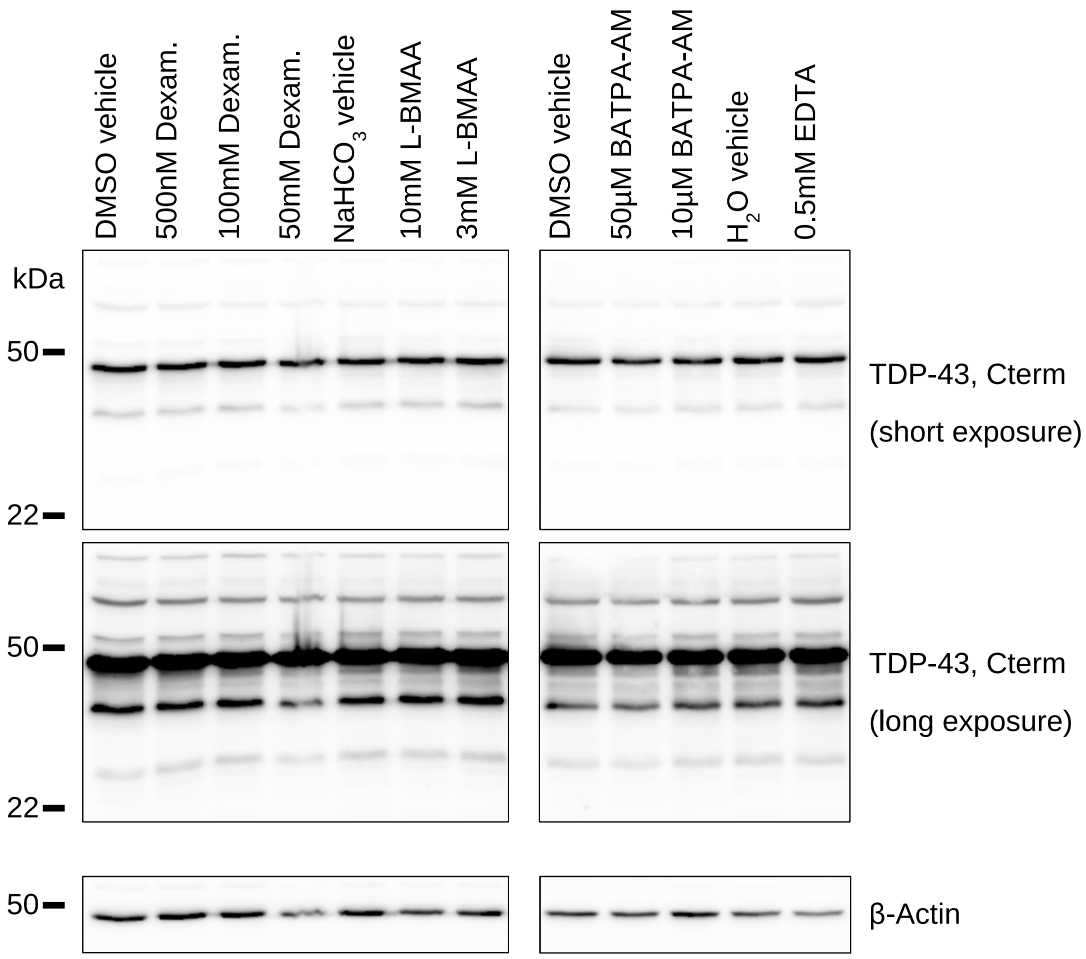

Western Blot analysis of UbG76V-GFP reporter cell line treated with different compounds reported to induce TDP-43 C-terminal fragmentation. UbG76V-GFP HEK293 cells were treated with the indicated compound or the respective vehicle control (DMSO for Dexamethasone and BAPTA-AM, NaHCO3 for L-BMAA or H2O for EDTA) for 24h. Total cell lysates were immunoblotted for TDP-43 using an anti-TDP-43 C-terminal antibody (proteintech #12892-1-AP). β-Actin served as a loading control.

Referee #2:

This is a well-written paper to use three pronged approaches of cryo-ET, mass spec and functional assays to tackle a complex neurodegeneration problem on where and how the TDP-25 impact on cellular functions. The conclusion that proteasome dysfunction is a hallmark of ALS/FTD pathogenesis is not surprising and opens more avenues in therapeutic strategies. However, I have a few technical questions on the cryoET analysis that needs to be clarified in the text or the supplement.

1. How do they pick the aggregates (i.e., the annotated red features) in Figure 1D to be GFP-TDP-25? They are variable sizes and shapes. Was a control done from cells without GFP-TDP-25?

We apologize for the brief description in the original manuscript. We added a small section describing the procedure in the result section and extended the legend for Fig. 1D in the revised manuscript. In general, the identification is based on correlative light-electron microscopy (CLEM). Cryo-FIB lamella preparation was always guided by the cryo-LM information: the lamella was only prepared in the region with GFP signal (additional detailed information can be found in the Methods section).

We now indicate in the legend of Fig. 1 that the irregular aggregate structures were approximately segmented using a threshold-based approach for visualization purposes. The perimeter of the aggregate was approximately defined visually as the contour of the amorphous aggregate.

Reaching sufficient resolution to determine proteasome conformation requires a high number of particles, which can be much more easily achieved in conditions where proteasomes are concentrated such as TDP-25 inclusions. Analyzing a comparable number of particles in control cells would require an enormous amount of data, and thus we refer to a previous study where such a dataset was collected (Asano et al. 2015).

However, we have determined proteasome concentration in non-transduced and GFP-transduced neurons (Guo et al. 2018), reaching values comparable with Asano's work. One tomogram of an non-transduced neuron is now shown in the new Figure EV1E to illustrate the typical proteasome concentration in control cells. No structures similar to TDP-25 inclusions were found in control cells.

2. Are the TDP-25 inclusions localized only in certain region of the cells?

We found the TDP-25 inclusions to be mainly localized in the neuronal soma. This is now indicated in the revised manuscript. Occasionally, smaller inclusions could be identified in dendrites, but they were too small to process in our CLEM/cET pipeline.

3. Is there any cryo-ET structural difference between the wildtype and mutant GFP-TDP-25 inclusions?

This is an interesting question. With the current resolution we could not detect any differences between wild-type and mutant GFP-TDP-25 inclusion as indicated in the revised manuscript.

4. I am concerned about using template to pick ribosomes, proteosomes and TRiC. Have they carried out independent approach to validate the picked proteosomes to be in the same structural state? Again, a side by side control experiment without TDP-25 present cell would be great.

This is a valid remark. We applied the same approach of template matching and proteasome averaging as in our previous work (Guo et al, Cell, 2018). In that study, we showed that our computational workflow can distinguish different proteasome conformations if they are present. In brief, to avoid the influence of the template induced bias, low-resolution (~40 Å in resolution)

templates were always used. Thus, the higher-resolution details visible in averages must originate from the data itself and not from reference bias. This process is illustrated below for ribosomes within our tomograms, showing the template (left) and averaging result (right). Proteasome averages also gained higher-resolution features, as the ground and substrate processing conformations could not be distinguished at the resolution of the template. Additional features not present in the template were also observed in the average, such as the extra densities shown in Fig. 1E.

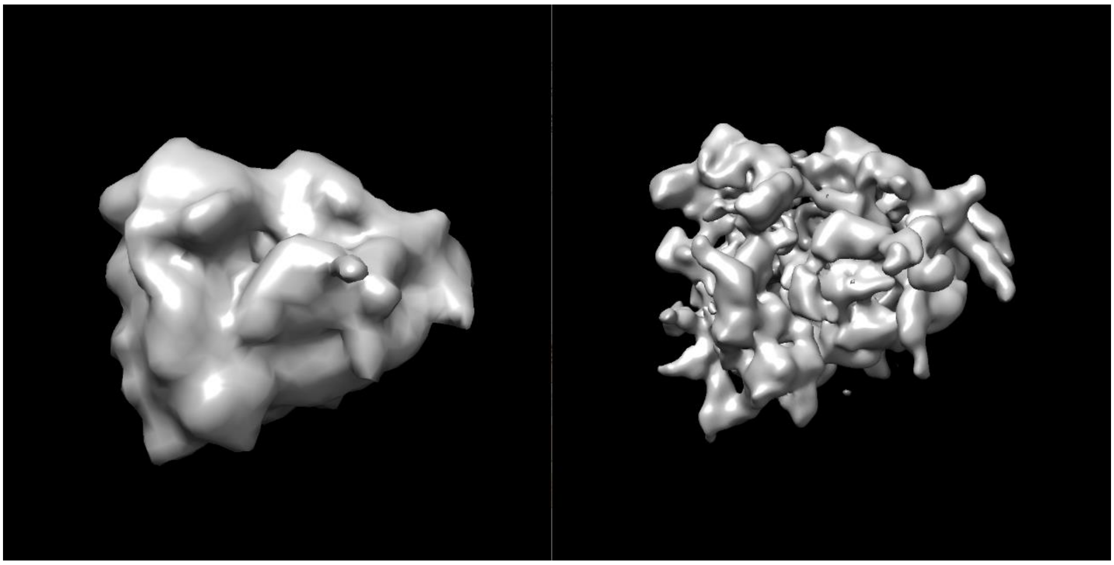

For the structural states analysis, all the subtomograms were used for multiple round of classification using RELION, which is a regularized likelihood based classification approach. The method is widely used in the field of cryo-EM/ET to separate conformationally heterogeneous particles. However, with this approach small subgroups with less than 10% of the total populations are difficult to separate. We have now added a sentence to the text acknowledging that a small fraction of proteasome particles in other conformations may be present.

5. What are the particle statistics to derive the plot in Supp Figure 1C?

In total 1723 proteasome caps were used for the final refinement as indicated in the revised legend. For the resolution estimation, we performed the gold-standard FSC calculation (Scheres, S. H, Nat. Methods, 2012): the subtomograms were separated randomly to two halves and perform refinement independently to get the two volumes for FSC calculation.

6. What are the cryoET data statistics before the conclusions are made. How do the plots shown in Supplement Figure 1D compared between cells without and with TDP-25 transduction.

The numbers of proteasome detected in the tomograms containing TDP-25 inclusions are listed below (average concentration = 1.6 µM, SD = 0.4 µM):

Tomogram	Tomogram volume (µm^3)	Number of proteasomes detected	Concentration (µM)
1	0.13	150	1.9
2	0.14	160	1.9
3	0.12	150	2.2
4	0.15	156	1.7
5	0.09	53	0.9

6	0.07	82	2.0
7	0.09	86	1.5
8	0.09	120	2.1
9	0.05	35	1.2
10	0.13	98	1.2
11	0.12	80	1.1

Note, that proteasome concentration was calculated for the whole tomographic volume, not only in the area occupied by the inclusion

7. It is necessary that at least one representative tomogram reconstruction is deposited to the EMDB for the public use. In addition, the subtomogram average of the proteasome along with the FSC plot should also be deposited.

We deposited the tomographic reconstruction shown in Fig 1C at EMDB with the accession code EMD-32217.

The subtomogram average of 26S proteasomes within neurons with TDP-25 inclusions has also been deposited at EMDB with the accession code EMD-32216.

Referee #3:

The manuscript by Riemenschneider et al. describes the characterisation of aggregation of a truncated version of TDP-25 in to inclusions. The paper reports the formation of solidified structures that appear to be non-fibrillar and associate with stalled conformation of proteosomes.

The paper is well written but the shortened format has lead to some questions regarding the approaches that are a unclear to the reader. The paper includes a large amount of electron tomography but the description of it really enough lacks detail. There is a lack of information on repeats, statistics and analysis methods.

We thank the reviewer for the interest in our study and the constructive comments. We expanded the description of the cryo-electron tomography section to make it easier comprehensible, but unfortunately, we are restricted by a tight word count. Moreover, we provide detailed statistical information for figures 2B and EV2.

Figure 1A shows a single example of co-localisation of TDP25 (wt or mutant) with P-TDP43. There is no quantification or statistics here. Figure 1B shows a single WB again with no quantification or stats.

We apologize for this oversight and added quantification for Fig 1A and B. Briefly, nearly all TDP-25 inclusions are phosphorylated at Serine 403/404 (TDP25-WT: 95.8 ± 2.9% vs TDP25-MUT: 96.2 ± 3.1%, mean ± SD from n=3 biological replicates, now mentioned in the revised result section). Quantification of the western blots shows that solubility of wild-type and mutant TDP-25 is similar (new Fig 1C).

In Figure 1C, how have TDP25 containing inclusions been identified in the cells? It would be helpful to include further explanation of the annotation process for ET. These additional details are necessary to give confidence for the reported results.

We added a small section describing the procedure in the result section and extended the legend for Fig. 1D in the revised manuscript. In general, the identification is based on correlative light-electron microscopy (CLEM). Cryo-FIB lamella preparation was always guided by the cryo-LM information: the lamella was only prepared in the region with GFP signal (additional detailed information can be found in the Methods section).

We have collected comparable datasets in control neurons, as well as cells harboring different inclusions (Bäuerlein et al, Cell 2017, Guo et al, Cell 2018, Trinkaus et al, Nat Comm 2021) and never found any similar structure. Altogether, we draw the conclusion that the condensed densities in the tomograms correspond to TDP-25 inclusions.

For the annotation, the inclusions were segmented by low-pass filtering and thresholding the higher intensity regions in Amira. The membranes were segmented with TomoSegMemTV software, with the missing region fixed manually in Amira. Molecules like proteasomes, ribosomes, TRiC were identified by template matching and pasted back with the position and Euler angles determined by template matching results (refinement star file in the case of proteasome). Additional information on these procedures has been included in the revised text.

Minor:

1. the abstract states that TDP43 correlates with FTD but in the introduction, it is more correctly stated that it correlates with 45% of FTD, so the abstract should be amended (some cases of FTD).

We adjusted the abstract in the revised manuscript accordingly.

2. This sentence in the introduction needs attention:

"Here, we aimed to elucidate gain-of-function mechanisms as well as the structure of cytoplasmic TDP-43 aggregates found in sporadic and most genetic ALS/FTD cases we focused on the aggregation-prone TDP-25 fragment (residues 220-414 of full length human TDP-43) (Neumann et al, 2006; Zhang et al, 2009) using a pipeline of cryo-ET, proteomics and functional assays"

We correct our grammar mistake.

Overall, this is an interesting paper but I feel unsure of the use and significance of the truncation version of TDP43. The authors may need to defend their use of this fragment further for the physiological relevance of their findings for ALS and FTD that involves TDP43

TDP-25 fragments are widely found in disease and considered as one of the major hallmarks of TDP-43 pathology, although they are clearly not the only component of the pathognomonic inclusions (Neumann et al, Science 2006). Per request of reviewer 1 we have analyzed the effect of full length TDP-43. Surprisingly, high expression levels of TDP-43 inhibit proteostasis (which is consistent with less pronounced proteasome recruitment revealed by mass spectrometry, see Fig 3A) in the absence of inclusion formation, and introducing eight ALS-causing mutations has an even bigger effect suggesting that our findings also have implications for full-length TDP-43.

Dear Dieter,

Thank you for the submission of your revised manuscript. We have now received the enclosed reports from the referees that were asked to assess it, and I am happy to say that both referees support the publication of your study now. We can therefore in principle accept it.

Only a few editorial requests still need to be addressed:

- Please upload all main and EV figures as separate, individual files.

- The Fig EV3A callout is missing, please add.

- Please upload the movie files as individual movies together with their legends in zipped files.

- Please make sure that all data deposited in public repositories are freely accessible to the readers upon the publication of this paper.

- The EV figure legends should be moved to after the main figure legends. Please also add a heading "Figure legends".

- I attach to this email a related ms file with comments by our data editors. Please address all comments in the final manuscript.

I would like to suggest a few minor changes to the abstract that needs to be written in present tense. I also modified a few sentences. Please check carefully and let me know whether anything is incorrect:

Aggregation of the multifunctional RNA-binding protein TDP-43 defines large subgroups of amyotrophic lateral sclerosis and frontotemporal dementia and correlates with neurodegeneration in both diseases. In disease, characteristic C-terminal fragments of ~25 kDa ("TDP-25") accumulate in cytoplasmic inclusions. Here, we analyze gain-of-function mechanisms of TDP-25 combining cryo-electron tomography, proteomics and functional assays. In neurons, cytoplasmic TDP-25 inclusions are amorphous, and photobleaching experiments reveal gel-like biophysical properties that are less dynamic than nuclear TDP-43 is. Compared with full length TDP-43, the TDP-25 interactome is depleted of low-complexity domain proteins. TDP-25 inclusions are enriched in 26S proteasomes in exclusively substrate-processing conformations, suggesting that inclusions sequester proteasomes that are largely stalled and no longer undergo the cyclic conformational changes required for proteolytic activity. Reporter assays confirm that TDP-25 impairs proteostasis, and this inhibitory function is enhanced by ALS-causing TDP-25 [or TDP-43??] mutations. These findings support a patho-physiological relevance of proteasome dysfunction in ALS/FTD.

I also slightly modified the short summary and bullet points. Do you agree with:

TDP-25, a C-terminal fragment of TDP-43 found in ALS and FTD patients, forms cytoplasmic inclusions with gel-like properties in primary neurons. Proteasome enrichment and impaired proteostasis support a relevance of proteasome dysfunction in ALS/FTD.

• neuronal cytoplasmic TDP-25 inclusions adopt an amorphous gel-like state without detectable fibrils

• stalled proteasomes are present in the inclusions

• multiple ALS-causing mutations further increase proteasomal impairment

I look forward to seeing a final version of your manuscript as soon as possible. Please use this link to submit your revision: https://embor.msubmit.net/cgi-bin/main.plex

Best regards,
Esther

Esther Schnapp, PhD
Senior Editor
EMBO reports

Referee #1:

Authors have answered very well all the major issues raised by this reviewer

Referee #3:

The revised paper is much improved having clarified some and addition of stats has given more weight to the result. Overall, I would have liked to see some additional information as the response letter gives a nice amount of detail but I understand that the paper has length restrictions.

Referee 1's comment on referee 2's concerns:

a) The first major concern of the reviewer was about the way aggregates were picked (basically a technical query). To this, the authors answered fully and better explained the procedure.
b) The second query was not really a concern but just the need for a clarification regarding the localization of the aggregates (and authors complied)
c) The third query was again just a curiosity: whether there were Cryo-ET differences between the WT and mutant proteins (there were not). Importantly, neither answer would have impacted the study or any further interpretation of the data.
d) The fourth query was a potentially major concern because it was not clear to this reviewer what had been the methodology to pick ribosomes, proteasome and TRiC. In my opinion, authors have also replied well to this query mentioning the fact that they used the same methodology that had been optimized in their 2018 Cell paper.
e) Queries 5 and 6 were also mostly technical, dealing with the need to provide additional CryoET statistics. I am not an expert here but to me the reply of the authors looks quite professional.
f) finally, query 7 was just a request to submit the structures to a data repository (which the authors did)

Overall, my feeling therefore is that the authors have complied with all requirements outlined by this reviewer.

The authors have addressed all minor editorial requests.

Prof. Dieter Edbauer
German Center for Neurodegenerative Diseases (DZNE) Munich
Cell Biology of Neurodegeneration
Feodor-Lynenstr. 17
Munich, Bavaria 81337
Germany

Dear Dieter,

I am very pleased to accept your manuscript for publication in the next available issue of EMBO reports. Thank you for your contribution to our journal.

Please note that under the DEAL agreement of German scientific institutions with our publisher Wiley, you could be eligible for publication of your article in the open access format in a way that is free of charge for the authors. Please contact either the administration at your institution or our publishers at Wiley (emboreports@wiley.com) for further questions.

At the end of this email I include important information about how to proceed. Please ensure that you take the time to read the information and complete and return the necessary forms to allow us to publish your manuscript as quickly as possible.

As part of the EMBO publication's Transparent Editorial Process, EMBO reports publishes online a Review Process File to accompany accepted manuscripts. As you are aware, this File will be published in conjunction with your paper and will include the referee reports, your point-by-point response and all pertinent correspondence relating to the manuscript.

If you do NOT want this File to be published, please inform the editorial office within 2 days, if you have not done so already, otherwise the File will be published by default [contact: emboreports@embo.org]. If you do opt out, the Review Process File link will point to the following statement: "No Review Process File is available with this article, as the authors have chosen not to make the review process public in this case."

Thank you again for your contribution to EMBO reports and congratulations on a successful publication. Please consider us again in the future for your most exciting work.

Best regards,
Esther

Esther Schnapp, PhD
Senior Editor
EMBO reports

****

THINGS TO DO NOW:

Please note that you will be contacted by Wiley Author Services to complete licensing and payment information. The required 'Page Charges Authorization Form' is available here: https://www.embopress.org/pb-assets/embo-site/er_apc.pdf

You will receive proofs by e-mail approximately 2-3 weeks after all relevant files have been sent to our Production Office; you should return your corrections within 2 days of receiving the proofs.

Please inform us if there is likely to be any difficulty in reaching you at the above address at that time. Failure to meet our deadlines may result in a delay of publication, or publication without your corrections.

All further communications concerning your paper should quote reference number EMBOR-2021-53890V3 and be addressed to emboreports@wiley.com.

Should you be planning a Press Release on your article, please get in contact with emboreports@wiley.com as early as possible, in order to coordinate publication and release dates.

EMBO PRESS

YOU MUST COMPLETE ALL CELLS WITH A PINK BACKGROUND

PLEASE NOTE THAT THIS CHECKLIST WILL BE PUBLISHED ALONGSIDE YOUR PAPER

Corresponding Author Name: Ruben Fernandez-Busnadiego, Dieter Edbauer

Journal Submitted to: EMBO Reports
Manuscript Number: EMBOR-2021-53890

Reporting Checklist For Life Sciences Articles (Rev. June 2017)

This checklist is used to ensure good reporting standards and to improve the reproducibility of published results. These guidelines are consistent with the Principles and Guidelines for Reporting Preclinical Research issued by the NIH in 2014. Please follow the journal's authorship guidelines in preparing your manuscript.

A- Figures
1. Data
The data shown in figures should satisfy the following conditions:

- the data were obtained and processed according to the field's best practice and are presented to reflect the results of the experiments in an accurate and unbiased manner.
- figure panels include only data points, measurements or observations that can be compared to each other in a scientifically meaningful way.
- graphs include clearly labeled error bars for independent experiments and sample sizes. Unless justified, error bars should not be shown for technical replicates.
- if n< 5, the individual data points from each experiment should be plotted and any statistical test employed should be justified
- Source Data should be included to report the data underlying graphs. Please follow the guidelines set out in the author ship guidelines on Data Presentation.

2. Captions

Each figure caption should contain the following information, for each panel where they are relevant:

- a specification of the experimental system investigated (eg cell line, species name).
- the assay(s) and method(s) used to carry out the reported observations and measurements
- an explicit mention of the biological and chemical entity(ies) that are being measured.
- an explicit mention of the biological and chemical entity(ies) that are altered/varied/perturbed in a controlled manner.

- the exact sample size (n) for each experimental group/condition, given as a number, not a range;
- a description of the sample collection allowing the reader to understand whether the samples represent technical or biological replicates (including how many animals, litters, cultures, etc.).
- a statement of how many times the experiment shown was independently replicated in the laboratory.
- definitions of statistical methods and measures:
  - common tests, such as t-test (please specify whether paired vs. unpaired), simple χ2 tests, Wilcoxon and Mann-Whitney tests, can be unambiguously identified by name only, but more complex techniques should be described in the methods section;
  - are tests one-sided or two-sided?
  - are there adjustments for multiple comparisons?
  - exact statistical test results, e.g., P values = x but not P values < x;
  - definition of 'center values' as median or average;
  - definition of error bars as s.d. or s.e.m.

Any descriptions too long for the figure legend should be included in the methods section and/or with the source data.

In the pink boxes below, please ensure that the answers to the following questions are reported in the manuscript itself. Every question should be answered. If the question is not relevant to your research, please write NA (non applicable). We encourage you to include a specific subsection in the methods section for statistics, reagents, animal models and human subjects.

USEFUL LINKS FOR COMPLETING THIS FORM

http://www.antibodypedia.com
http://1degreebio.org
http://www.equator-network.org/reporting-guidelines/improving-bioscience-research-repor

http://grants.nih.gov/grants/olaw/olaw.htm
http://www.mrc.ac.uk/Ourresearch/Ethicsresearchguidance/Useofanimals/index.htm
http://ClinicalTrials.gov
http://www.consort-statement.org
http://www.consort-statement.org/checklists/view/32-consort/66-title
http://www.equator-network.org/reporting-guidelines/reporting-recommendations-for-tumo
http://datadryad.org
http://figshare.com
http://www.ncbi.nlm.nih.gov/gap
http://www.ebi.ac.uk/ega
http://biomodels.net/
http://biomodels.net/miriam/
http://jjj.biochem.sun.ac.za
https://osp.od.nih.gov/biosafety-biosecurity-and-emerging-biotechnology/
http://www.selectagents.gov/

B- Statistics and general methods

Please fill out these boxes (Do not worry if you cannot see all your text once you press return)

1.a. How was the sample size chosen to ensure adequate power to detect a pre-specified effect size?	Due to the exploratory nature of our study, we could not run power analyses to pre-detrmine sample sizes. Each experiment including a quantification was performed independently at least three times.
1.b. For animal studies, include a statement about sample size estimate even if no statistical methods were used.	Not applicable
2. Describe inclusion/exclusion criteria if samples or animals were excluded from the analysis. Were the criteria pre-established?	One technical replicate for Fig1B (GFP-TDP-25 mut, biological replicate #2) had to be excluded due to inappropriate transfer during immuoblotting. Other than this, no samples were excluded.
3. Were any steps taken to minimize the effects of subjective bias when allocating animals/samples to treatment (e.g. randomization procedure)? If yes, please describe.	Wells for viral transduction and compound treatment were randomized to exclude edge effects during experiments.
For animal studies, include a statement about randomization even if no randomization was used.	Not applicable
4.a. Were any steps taken to minimize the effects of subjective bias during group allocation or/and when assessing results (e.g. blinding of the investigator)? If yes please describe.	Images were taken blinded and in a randomized fashion using tile scan mode if quantifications were performed. Fractionation and flow cytometry experiments were carried out blinded regarding the construct transduced or transfected.
4.b. For animal studies, include a statement about blinding even if no blinding was done	Not applicable
5. For every figure, are statistical tests justified as appropriate?	Commonly used statistical tests were applied if experiments were carried out independently at least three times. For most biological replicates, several technical replicates (individually transduced wells of the same primary neuron preparation) were analyzed and averaged. Statistical tests were applied only if the assumptions were met (see below).
Do the data meet the assumptions of the tests (e.g., normal distribution)? Describe any methods used to assess it.	Normal distribution as well as homogeneity of variances were assessed to choose between a parametric or non-parametric test. We used R to test for normal distribution via Shapiro–Wilk test and/or QQ plots. Levene's test and visual inspection of the residuals were used to test for equality of variances. Statistical comparisons were performed for more than two groups.
Is there an estimate of variation within each group of data?	Variations were indicated by reporting standard deviation (SD) and 95% confidence intervals (CI).

Is the variance similar between the groups that are being statistically compared?	Equality of variances was tested using Levene's Test prior to runnig a statistical test. If homogeneity of variances was violated, a non-parametric test was used.

C- Reagents

6. To show that antibodies were profiled for use in the system under study (assay and species), provide a citation, catalog number and/or clone number, supplementary information or reference to an antibody validation profile. e.g., Antibodypedia (see link list at top right), 1DegreeBio (see link list at top right).	GFP (UC Davis/NIH NeuroMab Facility, Cat# 75-131, clone ID N86/8, RRID:AB_10671445), GFP (UC Davis/NIH NeuroMab Facility, Cat# N86/38, clone ID N86/38, RRID:AB_2877499), Calnexin (Enzo Life Sciences, Cat# ADI-SPA-860, RRID:AB_10616095), Map2 (Sigma-Aldrich, Cat# M1406, clone ID AP-20, RRID:AB_477171), TDP-43, phospho Ser403/404 (Cosmo Bio Co., Ltd., Cat# TIP-PTD-P05)
7. Identify the source of cell lines and report if they were recently authenticated (e.g., by STR profiling) and tested for mycoplasma contamination.	HEK293FT (purchased from Thermo Fisher Scientific), UbG76V-GFP HEK293 stable cell line (DeSmet et al., 2017). All cell lines were regulary checked for mycoplasma contamination and tested negative.

* for all hyperlinks, please see the table at the top right of the document

D- Animal Models

8. Report species, strain, gender, age of animals and genetic modification status where applicable. Please detail housing and husbandry conditions and the source of animals.	Rats (IGS background, Charles River; RRID:RGD_734476) were housed in a pathogen-free facility with 12:12 h light/dark cycle and food/water available ad libitum.
9. For experiments involving live vertebrates, include a statement of compliance with ethical regulations and identify the committee(s) approving the experiments.	All animal experiments were performed in compliance with institutional policies approved by the government of Upper Bavaria following §11 Ab.1 TierSchG for the DZNE animal facility (Inst.-Nr. 04-26).
10. We recommend consulting the ARRIVE guidelines (see link list at top right) (PLoS Biol. 8(6), e1000412, 2010) to ensure that other relevant aspects of animal studies are adequately reported. See author guidelines, under 'Reporting Guidelines'. See also: NIH (see link list at top right) and MRC (see link list at top right) recommendations. Please confirm compliance.	Not applicable

E- Human Subjects

11. Identify the committee(s) approving the study protocol.	Not applicable
12. Include a statement confirming that informed consent was obtained from all subjects and that the experiments conformed to the principles set out in the WMA Declaration of Helsinki and the Department of Health and Human Services Belmont Report.	Not applicable
13. For publication of patient photos, include a statement confirming that consent to publish was obtained.	Not applicable
14. Report any restrictions on the availability (and/or on the use) of human data or samples.	Not applicable
15. Report the clinical trial registration number (at ClinicalTrials.gov or equivalent), where applicable.	Not applicable
16. For phase II and III randomized controlled trials, please refer to the CONSORT flow diagram (see link list at top right) and submit the CONSORT checklist (see link list at top right) with your submission. See author guidelines, under 'Reporting Guidelines'. Please confirm you have submitted this list.	Not applicable
17. For tumor marker prognostic studies, we recommend that you follow the REMARK reporting guidelines (see link list at top right). See author guidelines, under 'Reporting Guidelines'. Please confirm you have followed these guidelines.	Not applicable

F- Data Accessibility

18: Provide a "Data Availability" section at the end of the Materials & Methods, listing the accession codes for data generated in this study and deposited in a public database (e.g. RNA-Seq data: Gene Expression Omnibus GSE39462, Proteomics data: PRIDE PXD000208 etc.) Please refer to our author guidelines for 'Data Deposition'.<br><br>Data deposition in a public repository is mandatory for:<br>a. Protein, DNA and RNA sequences<br>b. Macromolecular structures<br>c. Crystallographic data for small molecules<br>d. Functional genomics data<br>e. Proteomics and molecular interactions	Protein interaction AP-MS data: PRIDE PXD024358<br>cryoET Imaging Dataset: EMDB EMD-32216, EMD-32217
19. Deposition is strongly recommended for any datasets that are central and integral to the study; please consider the journal's data policy. If no structured public repository exists for a given data type, we encourage the provision of datasets in the manuscript as a Supplementary Document (see author guidelines under 'Expanded View' or in unstructured repositories such as Dryad (see link list at top right) or Figshare (see link list at top right).	Not applicable
20. Access to human clinical and genomic datasets should be provided with as few restrictions as possible while respecting ethical obligations to the patients and relevant medical and legal issues. If practically possible and compatible with the individual consent agreement used in the study, such data should be deposited in one of the major public access-controlled repositories such as dbGAP (see link list at top right) or EGA (see link list at top right).	Not applicable
21. Computational models that are central and integral to a study should be shared without restrictions and provided in a machine-readable form. The relevant accession numbers or links should be provided. When possible, standardized format (SBML, CellML) should be used instead of scripts (e.g. MATLAB). Authors are strongly encouraged to follow the MIRIAM guidelines (see link list at top right) and deposit their model in a public database such as Biomodels (see link list at top right) or JWS Online (see link list at top right). If computer source code is provided with the paper, it should be deposited in a public repository or included in supplementary information.	Not applicable

G- Dual use research of concern

22. Could your study fall under dual use research restrictions? Please check biosecurity documents (see link list at top right) and list of select agents and toxins (APHIS/CDC) (see link list at top right). According to our biosecurity guidelines, provide a statement only if it could.	Not applicable
